# Automatic Segmentation of Ischaemic Stroke Lesions Using Transformers and Convolutional Neural Networks Applied to Multimodal Neuroimaging

**DOI:** 10.3390/s26165021

**Published:** 2026-08-07

**Authors:** Pablo Martínez Cegarra, Juan Francisco Zapata Pérez, Juan Martínez-Alajarín

**Affiliations:** Escuela Técnica Superior de Ingeniería Industrial, Campus Muralla del Mar, Universidad Politécnica de Cartagena, C/Doctor Fleming, s/n, 30202 Cartagena, Spain; juan.zapata@upct.es (J.F.Z.P.); juanc.martinez@upct.es (J.M.-A.)

**Keywords:** ischaemic stroke, deep learning, Transformers, multimodal imaging, automated segmentation, ISLES 2024

## Abstract

Ischaemic stroke constitutes a leading cause of global disability. Rapid extraction of the infarct core from multimodal computed tomography perfusion (CTP) imaging guides reperfusion therapy and clinical decision-making. Deep learning algorithms automate this delineation, yet hospital translation is hindered by high-dimensional data, inter-scanner variability, and the low contrast of early ischaemia. Architectural comparisons in the literature frequently carry methodological biases originating from disparate preprocessing protocols and data partitions. This study reduces these variables by evaluating three segmentation strategies under a shared preprocessing pipeline and an identical data partition using the ISLES 2024 dataset. Three models were trained on the same 133-patient partition using a shared preprocessing pipeline based on morphological skull-stripping and modality-specific clinical intensity ranges. The data, preprocessing, and partitions are held constant across models, while framework-dependent factors (optimiser, patch size, physical field of view, spatial resampling, augmentation policy, and model capacity) remain coupled to each architecture and are therefore treated as part of the compared strategy rather than as fully isolated variables. The first of these is a single-stage 5-channel nnU-Net, followed by a two-stage cascaded nnU-Net (2 and 7 channels) and a lightweight Transformer (SegFormer3D). Evaluation on a fixed 15-patient held-out test set isolated the architectural performance. The cascade model achieved the highest Dice Similarity Coefficient (0.224). The single-stage nnU-Net provided the most precise volumetric estimation, recording an Absolute Volume Difference (AVD) of 23.70 mL and a lesion-wise F1-score of 7.60%. On the other hand, SegFormer3D returned the lowest overall metrics (DSC 0.163, AVD 27.28 mL, F1 2.30%). In the small held-out cohort, paired statistical testing did not reveal significant differences between models, so the reported orderings describe the present dataset and experimental configuration rather than a general architectural law. Within these limits, the local inductive bias of the convolutional models retained an empirical advantage over the single Transformer evaluated when processing this moderately sized neuroimaging dataset, and complex cascade topologies offered only marginal gains compared with a well-calibrated single-stage network. Although the predictive segmentation of infarcted tissue at acute stages still demands computational improvements, these results suggest that preprocessing quality is at least as decisive for clinical impact as increasing the complexity of neural architectures.

## 1. Introduction

Ischaemic stroke is one of the leading causes of death and permanent neurological disability worldwide, accounting for approximately 87% of all stroke cases [1]. Rapid and accurate delineation of the ischaemic lesion volume is of vital importance in clinical decision-making, as it informs reperfusion therapy selection, guides surgical planning, and helps predict the patient’s functional outcome [2]. In current clinical practice, lesion segmentation is performed manually by expert neuroradiologists, a process that is time-consuming and prone to inter-observer variability. Scaling it to the case volumes demanded by modern stroke units remains an open clinical challenge.

Multimodal computed tomography perfusion (CTP) imaging provides complementary information about cerebral haemodynamics through maps of cerebral blood flow (CBF), cerebral blood volume (CBV), mean transit time (MTT), and time-to-maximum (Tmax), alongside non-contrast CT (NCCT) and CT angiography (CTA). The joint use of these modalities offers the possibility of more easily distinguishing the ischaemic core from the penumbra, and provides an opportunity to improve segmentation accuracy beyond what could be achieved with a single modality [3]. However, the high dimensionality of multimodal volumetric data, the inter-site acquisition variability inherent to multi-centre datasets, and the severe class imbalance between lesion and healthy tissue voxels pose substantial challenges for automated methods.

Convolutional neural networks (CNNs) dominate current medical image segmentation due to their good results [4]. Among CNN-based frameworks, nnU-Net stands out for its self-configuring pipeline, which automatically adapts network architecture, preprocessing, and training schedule to each dataset without manual intervention [5]. More recently, Vision Transformer (ViT) architectures have introduced global self-attention mechanisms as an alternative to local convolutions, enabling the capture of long-range spatial dependencies and characteristics across the entire volume [6]. Research on their adaptation to volumetric medical image segmentation remains open: global attention in Transformers requires considerably larger datasets to learn meaningful representations, and the fixed window-size constraints of early Transformer designs are poorly suited to the heterogeneous volume sizes encountered in neuroimaging.

The Ischemic Stroke Lesion Segmentation Challenge 2024 (ISLES’24) provided a standardised benchmark for this problem, releasing a multimodal CTP dataset of 150 annotated training cases from multiple clinical centres, together with an independent 100-patient test set evaluated on a hidden leaderboard [7]. The top-ranked submission demonstrated that a carefully designed preprocessing pipeline—including skull-stripping and modality-specific intensity windowing—combined with nnU-Net in its 3D full-resolution configuration yielded a mean Dice similarity coefficient (DSC) of 0.285 on the official test set [8]. All three top-ranked submissions relied on variants of nnU-Net. Transformer-based approaches, by contrast, showed markedly lower performance; the best Transformer entry achieved a DSC of only 0.068 [7].

The main limitation of drawing conclusions from comparisons derived from the challenge leaderboard is that the results of the competing strategies are subject to confounding variables such as differences in preprocessing protocols, training data volume, and cross-validation strategy, making it difficult to attribute performance gaps to architectural choice alone.

Because of that, in this work we seek to close this gap by training, evaluating, and comparing three distinct deep learning strategies for ischaemic stroke lesion segmentation on the ISLES’24 dataset under strictly controlled experimental conditions: (i) a single-stage nnU-Net approach replicating the preprocessing pipeline of the challenge-winning team under controlled experimental conditions, using 5 input channels (CTA, CBF, CBV, MTT, Tmax); (ii) a two-stage cascade approach combining the same preprocessing strategy with the multi-channel fusion scheme of the second-ranked team, where a preliminary segmentation mask is generated from 2 channels and subsequently injected as an additional input to a 7-channel model; and (iii) a SegFormer3D-based approach, a lightweight pure Transformer architecture adapted for volumetric segmentation without fixed window-size constraints, trained with identical preprocessing. All three strategies are trained on the same subset of 106 patients, with a validation fold of 27 patients, evaluated on the same 15-patient held-out test set, and assessed using the four official ISLES’24 metrics: DSC, Absolute Volume Difference (AVD), lesion-level F1-score, and Absolute Lesion Count Difference (ALCD).

The main contributions of this work are:

- A controlled experimental comparison of CNN and Transformer segmentation strategies for 3D multimodal ischaemic stroke segmentation under a shared preprocessing pipeline and an identical data partition, explicitly distinguishing the variables that are held constant (data, preprocessing, splits) from the framework-dependent factors that remain coupled to each architecture (optimiser, patch size, field of view, resampling, augmentation and model capacity).
- A methodologically controlled replication of the ISLES’24 challenge-winning pipeline, incorporating explicit skull-stripping and a fixed held-out test set to enable fair cross-model comparison.
- A quantitative analysis of two-stage cascade segmentation, evaluating whether incorporating the prediction of a first CNN model—trained on only 2 channels—as an additional input channel in a second 7-channel stage produces significant performance improvements on a fixed test set, or whether the increase in input dimensionality does not translate into a real gain over the single-stage model.
- Empirical evidence that applying an appropriate preprocessing pipeline to a Transformer architecture (SegFormer3D) yields results superior to those of Transformer-based submissions in the official challenge, suggesting that preprocessing quality may be a key factor exerting greater influence on performance than the choice of an apparently more powerful architecture trained under a different preprocessing scheme.

In short, the novelty of this study lies in three points: (i) a like-for-like comparison of CNN and Transformer segmentation strategies on ISLES’24 in which the data, the preprocessing and the partitions are fixed, so that the remaining differences reflect the training strategy of each framework as a whole; (ii) a quantitative test of whether a two-stage cascade justifies its added cost over a single-stage network on this task; and (iii) evidence that a lightweight Transformer trained with a clinically motivated preprocessing pipeline improves on the Transformer-based entries of the official challenge, which points to preprocessing as a decisive design choice. All comparisons are accompanied by paired statistical analysis and are interpreted as findings specific to this dataset and configuration.

The remainder of this paper is organised as follows: Section 2 reviews related work on deep learning for stroke lesion segmentation and the evolution of CNN and Transformer architectures in medical imaging. The dataset, preprocessing pipeline, model architectures, and evaluation protocol are described in Section 3. Section 4 presents the quantitative results for all three strategies. Finally, Section 5 discusses the findings in the context of the challenge literature and clinical applicability, and Section 6 summarises the main conclusions and outlines future research directions.

## 2. Related Work

### 2.1. From Atlas-Based Methods to Convolutional Neural Networks

Initially, the first strategies for brain lesion segmentation were based on the use of atlas registration. In this type of approach, a healthy brain was taken as a reference, which was spatially aligned with the volume of the patient’s brain. Subsequently, a voxel-by-voxel comparison was performed, and the differences from this comparison were defined as the pathological tissue. The problem with these methods is the dependence on a non-rigid registration, which gets worse when large lesions distort the anatomy or when the variability between patients is high. Probabilistic atlases were subsequently used as a partial solution to this problem. The strategy of these probabilistic atlases consisted of encoding population-level priors, but their performance remained bounded by the quality of the registration and the representativeness of the atlas population [9].

The transition to learning-based methods began with the use of random forests applied to handcrafted voxel features extracted from multi-sequence MRI [10]. Random forests dominated the first ISLES edition in 2015 [10] by achieving competitive results through meticulous feature engineering. The main problem of this strategy was the inability to learn spatial context beyond the local patch, restricting in this way the segmentation coherence across the volume.

This patch-level bottleneck was subsequently eliminated by fully convolutional networks (FCNs) [11], which generated dense pixel-by-pixel predictions in a single forward pass, with Ronneberger et al. being among those responsible for extending the FCN paradigm to biomedical images with U-Net [12]. They did this by introducing skip connections between the encoder and the decoder to preserve spatial resolution during upsampling. U-Net became one of the leading architectures in medical image segmentation. The development of this architecture led to its adaptation to volumetric data with the use of 3D U-Net [13], which replaced 2D convolutions with their 3D counterparts, and finally, V-Net [14] introduced the Dice loss function, optimising in this way the overlap metric used in evaluation.

As architectures became deeper, residual connections [15] allowed networks with more than 100 layers to be trained without gradient degradation. DeepMedic [16] combined dual-pathway multi-scale processing with post-processing through conditional random fields (CRF), reaching the state of the art in brain tumour and lesion segmentation tasks. Attention mechanisms were subsequently incorporated into the U-Net decoder by Oktay et al. [17], who proposed Attention U-Net to selectively amplify features in regions of greater interest while suppressing irrelevant background activations.

### 2.2. Limitations of CNNs and the Rise of Vision Transformers

The main limitation of convolutional architectures compared with Transformers is that they are structurally designed to process local information. This is because each convolutional kernel operates on a fixed spatial neighbourhood, and the expansion of the receptive fields occurs through the stacking of layers or dilated convolutions, creating a local bias that, for brain lesion segmentation, can limit the network’s capacity to take into account information from distant anatomical structures, such as the comparison of perfusion values from a potentially affected hemisphere with those from the healthy one. An increase in depth or in dilation factors could result in an enlargement of the theoretical receptive field, but on the other hand, the effective receptive field remains concentrated around the centre of the kernel [18].

A solution to this was proposed, in part, by Vaswani et al. [19] with the Transformer architecture. Its operation consists of processing sequences by computing, for each element, how much attention it should pay to all the others, regardless of their positions. This method allows long-range dependencies to be calculated, something that CNNs cannot do directly. This mechanism was transferred to images with the Vision Transformer (ViT) by Dosovitskiy et al. [6], by dividing the image into non-overlapping patches of fixed size and subsequently converting them into numerical vectors. The Transformer processes this sequence as if the vectors were words, providing the main advantage of this type of architecture: any region of the image can be related to any other. On the other hand, it has to be highlighted that this has a cost, and the reason is that CNNs have the advantage of assuming by design that nearby pixels are related, whereas ViT does not assume this and needs to learn those relationships from the data, which translates into the need to train with larger datasets to obtain competitive performance on ImageNet classification.

The main problem of applying ViT to medical image segmentation is its quadratic complexity $O(n^{2})$, which makes the direct application to high-resolution 3D volumes computationally prohibitive. Hybrid architectures addressed this in two ways: the first combined Transformer encoders with CNN decoders, such as TransUNet [20], which tokenised CNN feature maps before processing them with Transformer layers, and UNETR [21], which replaced the entire encoder with a ViT connected to a CNN decoder through skip connections. The second reduced the computational cost from quadratic to linear through window-based attention, such as Swin Transformer [22] which computed attention locally in non-overlapping windows shifted between layers, a strategy which Swin UNETR [23] subsequently extended to 3D volumes, and on which nnFormer [24], TransBTS [25] and TransFuse [26] were built with different variants of CNN-Transformer fusion. The cost of this approach lies in the size of these architectures, since they require between 62 and 150 million parameters, which limits their use in clinical settings with scarce data or restricted hardware.

To overcome this limitation, SegFormer [27] proposed a hierarchical Transformer encoder with an all-MLP decoder, eliminating in this way the overhead of complex decoders without sacrificing multi-scale representation. The architecture was subsequently adapted to volumetric data with SegFormer3D [28], which achieved competitive results on BraTS, Synapse, and ACDC with only 4.5 million parameters and 17 GFLOPS, a reduction of 34× in parameters and 13× in computational cost compared with nnFormer (150.5 M parameters, 213.4 GFLOPS). In addition, it should be noted that its position-encoding-free design eliminates the fixed input size constraints, making it directly applicable to heterogeneous neuroimaging volumes without prior resizing. Recent reviews of deep learning for medical image segmentation confirm that the balance between CNN-based, Transformer-based and hybrid designs remains dataset-dependent, with no single family dominating across all tasks [29,30].

### 2.3. The ISLES Challenge Series (2015–2024)

The ISLES challenge has provided standardised benchmarks for stroke image analysis since 2015, with editions hosted at five MICCAI conferences [7]. Each edition redefined the task to address a different clinical question, progressively shifting from MRI-based retrospective analysis toward CT-based prospective prediction.

ISLES’15 [10] was based on the segmentation of sub-acute ischaemic stroke lesions on post-interventional MRI, and on the segmentation of acute perfusion lesions on pre-interventional MRI, with random forests with handcrafted features dominating at that time, although the paradigm shift was already being anticipated with the beginning of the use of CNN-based solutions, which would consolidate in the following editions. ISLES’16 and ISLES’17 [31] had as their objective the prediction of clinical outcomes, requiring the participants to predict patient disability scores by means of the use of follow-up stroke lesions from acute multimodal MR imaging. ISLES’22 [32] expanded the scope to more than 2000 MRI scans covering acute, sub-acute and chronic ischaemic stroke. Beyond the challenge series, recent work has continued to explore CT-perfusion lesion segmentation with dedicated deep architectures, such as dense residual encoder–decoder networks operating on multiparametric CTP maps [33] and attention-based residual U-Nets combining edge-enhancing preprocessing with modified decoders [34], underlining that preprocessing and network design are still actively co-optimised for this modality.

ISLES’24 [7] represents a paradigm shift in the evolution of the challenge. The models developed by the participants had to predict the final post-treatment infarct from acute pre-interventional CT images instead of segmenting visible pathology, which results in a notable extra complication for the models. The dataset is composed of 248 cases in total, of which 98 were reserved for testing (they were not made available to the teams) and 150 for training, originating from two clinical centres, University Hospital of Munich and University Hospital of Zurich. It should be noted that the dataset is restricted to patients with successful reperfusion (TICI 2B or 3), which excludes scenarios of failed or partial reperfusion and limits the generalisation of the models to real clinical practice. The input data consist of multimodal CT imaging acquired at admission (NCCT, CTA, raw 4D CTP sequence and its derived perfusion maps: CBF, CBV, MTT, Tmax), together with clinical data (demographic data, NIHSS at admission, 90-day mRS, among others). The ground truth masks were derived from follow-up MRI (DWI and ADC) acquired 2–9 days after admission, reviewed and corrected by neuroradiologists with more than 10 years of experience. The evaluation was performed on the hidden test set using four metrics: DSC, AVD, lesion-wise F1-score (IoU ≥ 20%), and ALCD.

The three top-ranked teams in ISLES’24 used variants of nnU-Net [7]. The winning team (Kurtlab) achieved a mean DSC of 0.285 ± 0.213 using a 3D nnU-Net with a large residual encoder and a custom preprocessing pipeline that included skull-stripping with SynthStrip [35] and modality-specific intensity windowing [8]. The second team (AMC-Axolotls) employed a two-stage cascaded nnU-Net, achieving a DSC of 0.263 ± 0.247. The third team (Ninjas) used the 4D CTP sequence exclusively as input and obtained a DSC of 0.255 ± 0.191. Transformer-based solutions performed substantially worse. The best hybrid CNN-Transformer entry (MIPLAB-PrediCTP), based on a 2D spatio-temporal network from Amador et al. [36], achieved a DSC of 0.139, and the only pure Transformer entry (QuiiL, using the MoReT architecture) obtained 0.068 [7].

### 2.4. nnU-Net and MONAI as Reference Frameworks

The nnU-Net framework [5] automates the design decisions that have traditionally required configuration by the designer, such as internal preprocessing, network topology (2D, 3D full-resolution or cascade), the training schedule and the post-processing. Given a new dataset, it analyses fingerprint statistics such as voxel spacing, image size distribution and class frequencies and configures the pipeline accordingly, matching or exceeding purpose-built architectures on 14 out of 17 benchmarks of the Medical Segmentation Decathlon [37].

Its dominance in ISLES’24 was not casual. Ren et al. [8] demonstrated that the gap between the standard pipeline and their winning solution was attributable to preprocessing, not to the architecture: standard Z-score normalisation gave a DSC of 21.8%, modality-specific windowing raised it to 31.0%, and adding histogram equalisation increased it to 31.8.

MONAI  [38] complements this ecosystem by offering modular components on top of PyTorch, without auto-configuration, which makes it the standard option for training non-nnU-Net architectures such as UNETR, Swin UNETR or SegFormer3D where flexibility in data loading and data augmentation is required.

### 2.5. Open Challenges: Small, Diffuse, and Evolving Lesions

The results of ISLES’24 demonstrate the difficulty of the challenge; the best DSC obtained was 0.285, far below DSC values reached on pure segmentation benchmarks such as BraTS (0.8–0.9) [39]. This gap is attributed to the predictive nature of the problem, since the model must predict tissue that is not yet visible on the admission CT, given that infarct evolution depends on collateral circulation and the response to reperfusion treatment [7], factors that cannot be appreciated from the input image. The temporal difference between the input and the ground truth (DWI at 2–9 days) imposes an extra difficulty that does not exist in conventional segmentation tasks.

In addition, there are technical difficulties inherent to the modality, since ischaemic lesions on CT show a much lower contrast than tumours on MRI due to the fact that early changes on NCCT manifest as subtle hypodensity, and CTP maps introduce noise and inter-scanner variability that force the models to rely on preprocessing to amplify the signal [8]. It should be added that lesion size and morphology present great variability, ranging from infarcts larger than 100 mL to others smaller than 1 mL (which tend to be undersegmented by the models), contributing in this way minimally to loss functions based on Dice [40], leading to the addition of the lesion-wise F1 metric with an IoU ≥ 20% threshold in ISLES’24.

These challenges are the main motivation for the experimental comparison presented in this work. If global self-attention enables Transformers to capture long-range cues such as hemispheric asymmetry in perfusion, the CNN-Transformer gap could narrow under controlled conditions. Otherwise, the results would constitute empirical evidence of the limits of Transformers in volumetric neuroimaging with moderate-sized datasets.

## 3. Materials and Methods

This section describes the dataset, the patient partition, the shared preprocessing pipeline for the three developed models, the architectures of the three models, the training strategy, and the evaluation metrics. The principle on which this research is based is that the data, the preprocessing and the training, validation and test partitions are kept identical across all models, so that these sources of variability do not drive the observed differences. It must be stated explicitly that the two frameworks used (nnU-Net for M1 and M2, MONAI/SegFormer3D for M3) do not share every training-time setting: optimiser (SGD versus AdamW), learning rate, patch size and physical field of view, spatial resampling, foreground sampling, validation batch size, inference procedure, augmentation policy and model capacity (∼30 M versus 4.5 M parameters) remain coupled to each architecture. These factors are therefore part of the strategy being compared rather than fully isolated variables, and this distinction is carried through the interpretation of the results (Section 5.3).

### 3.1. Dataset

The ISLES’24 dataset [7] contains 248 cases of acute ischaemic stroke acquired from two clinical centres. These centres are the University Hospital of Munich and the University Hospital of Zurich. Of these 248 cases, 150 were made public for the challenge, forming the public training set (100 from Munich, 50 from Zurich), and the remaining 98 were kept hidden as they were used to evaluate the teams that participated in the challenge. In this work, only the public training dataset is used.

The inclusion criteria in the challenge dataset are restricted to patients with large-vessel occlusion treated by endovascular thrombectomy with successful recanalisation (TICI score 2B or 3) [7]. Due to this, patients whose recanalisation was partial or failed, as well as those who did not receive interventional treatment, are excluded from the study, which indicates that the results are not directly extrapolable to patients with ischaemic stroke in general, but to a specific clinical subgroup with a relatively favourable prognosis.

**Figure 1:**
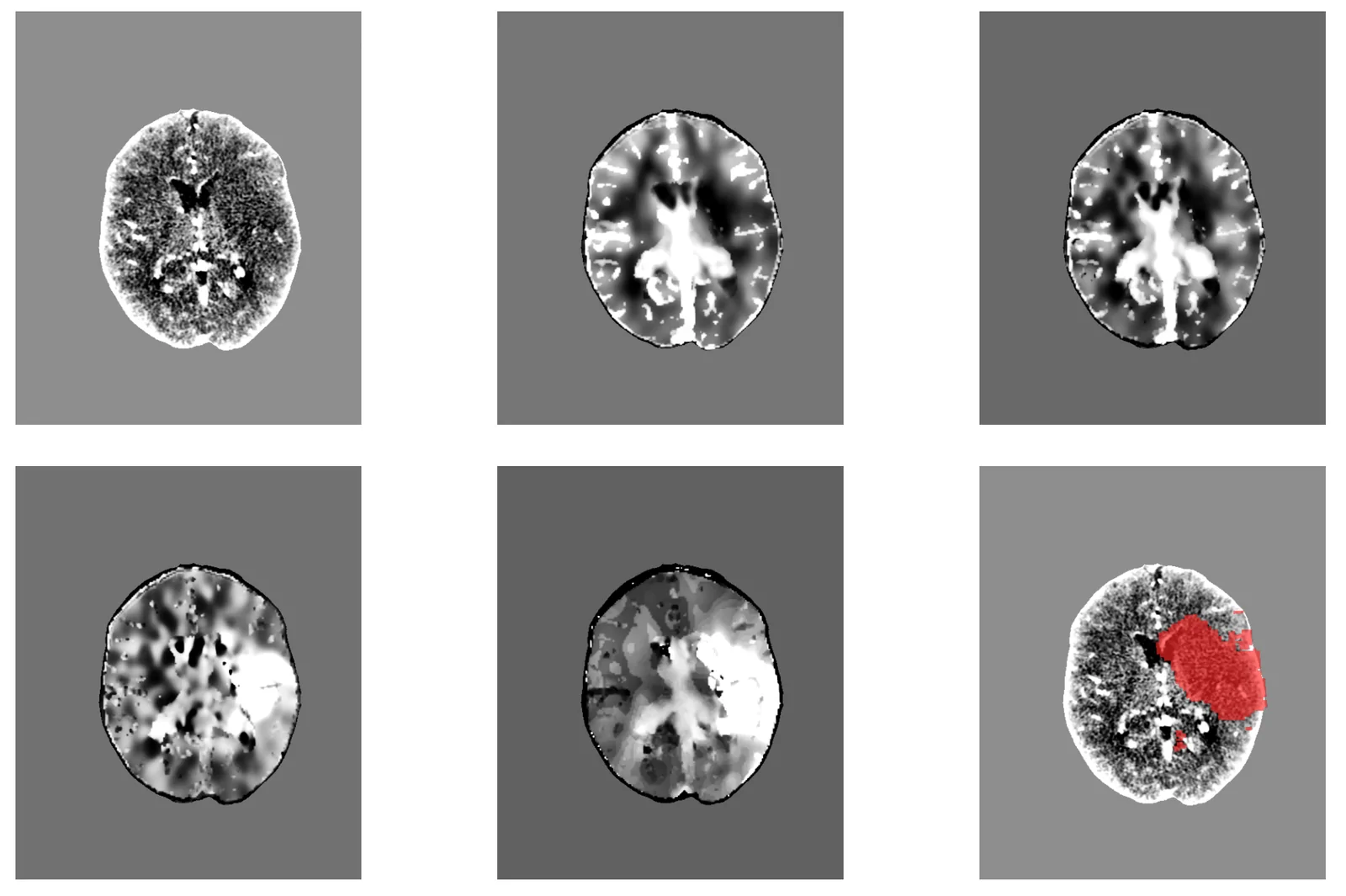
Representative case from the ISLES’24 training set after preprocessing (sub-stroke0027, axial slice z=30, lesion voxels =16,901). The figure is arranged in two rows and three columns. (**Top row**), from left to right: CTA, CBF, and CBV. (**Bottom row**), from left to right: MTT, Tmax, and CTA overlaid with the ground-truth infarct mask (red region). Each panel shows the same axial slice for the five input modalities used by M1 and M3, together with the ground-truth infarct mask derived from follow-up DWI.

Each case provides three pre-interventional CT modalities: *non-contrast CT* (NCCT), *CT angiography* (CTA), and *4D CT perfusion* (CTP). From the CTP, four derived perfusion maps are obtained by deconvolution: *cerebral blood flow* (CBF), *cerebral blood volume* (CBV), *mean transit time* (MTT) and *time-to-maximum* (Tmax). In addition to the images, a comprehensive set of tabular clinical data is included, covering patient demographics, medical history, laboratory results, neurological severity scores at admission (NIHSS scale) and functional outcomes at 3 months (mRS disability grade). *Diffusion-weighted MRI* (DWI) and *apparent diffusion coefficient* (ADC) maps are also provided for the final infarct segmentation, which was segmented semi-automatically by the DeepISLES algorithm and corrected by certified neuroradiologists with more than 10 years of experience [7]. It is worth noting the duality between the raw_data and derivatives branches: while raw preserves the original scanner images, the derivatives branch contains volumes that have been interpolated and mathematically aligned (co-registered) so that they fit onto the anatomical base of the NCCT [7]. Voxel resolution is heterogeneous; the NCCT offers the highest in-plane resolution (around 0.5×0.5 mm), while the CTP maps are coarser (≈1 × 1 mm), with slice thickness between 1 and 5 mm, so the volumes are not isotropic. Table 1 summarises the dataset. A representative case is shown in Figure 1.

### 3.2. Patient Partitioning

Of the 150 cases of the public dataset, 2 were discarded due to errors when reading certain channels, possibly due to the preparation of a smaller, cleaner dataset for Google Colaboratory. Of the remaining 148 patients, 15 were reserved for the internal test dataset, which were not seen by any of the 3 models at any time, and were chosen randomly. The remaining 133 patients were divided into 106 training patients and 27 validation patients, with the partition being identical for all models, thus guaranteeing the same train, validation, and test split for all models.

This scheme differs from the methodology of the challenge, where most teams trained on the 150 patients with cross-validation and inference was done on the set of 98 patients hidden by the organisation. The result is a decrease in statistical power, but the effect of the chosen architecture or model is isolated.

### 3.3. Preprocessing Pipeline

A single preprocessing pipeline is applied identically to all patients and developed models. This combines the windowing strategy of the challenge-winning team (Kurtlab) [8] with the additional NCCT channel of the second-ranked team (AMC-Axolotls) [7], replacing Kurtlab’s global histogram equalisation with a 3D *contrast-limited adaptive equalisation* (CLAHE) [41]. The complete sequence consists of five steps explained below.

First, morphological skull-stripping is applied to the NCCT of each patient by thresholding to (0,120) HU, binary erosion (3 iterations), selection of the largest 3D connected component with the cc3d library [42], dilation (5 iterations) and filling of internal holes. The resulting mask is propagated to the rest of the modalities by element-wise multiplication. SynthStrip [35], used by Kurtlab, proved unstable in the available computing environment. Visual inspection of a representative subset of cases confirmed the reliability of the generated morphological masks. The automated pipeline operated successfully, and no case in the held-out test set required exclusion or manual correction for gross skull-stripping failures. However, mild residual inclusion of non-brain voxels near the skull base cannot be ruled out; this is a known limitation of threshold-and-morphology approaches relative to learning-based tools such as SynthStrip.

Afterwards, modality-specific windowing is applied: each channel is clipped to its specific clinical range before any normalisation. The bounds follow the Kurtlab configuration for the CTP maps [8], with NCCT at (0,80) HU (standard stroke clinical window), narrower than the (0,100) HU of AMC-Axolotls.

Subsequently, min–max normalisation and CLAHE are applied. After windowing, each channel is rescaled to [0,1] using the modality-specific bounds reported in Table 2. Then, 3D CLAHE is applied (equalize_adapthist from scikit-image, clip limit 0.02, default kernel size), redistributing intensities within local neighbourhoods of the volume to enhance regional contrast while limiting noise amplification in homogeneous areas [41]. The operation is applied volumetrically over the native voxel grid; because the volumes are anisotropic (Z:XY spacing ratio ≈5:1), the equalisation kernel spans a larger physical extent along the axial direction than in-plane. The same clip limit is used identically for all channels and patients. Since CLAHE is a monotonic, locally order-preserving contrast operation and it is applied after modality-specific windowing and before foreground Z-score normalisation, it redistributes contrast within the retained clinical range rather than altering the relative ordering of tissue intensities; its effect on the perfusion-derived channels (CBF, CBV, MTT, Tmax) is examined in Section 5.1.

The next step applies foreground Z-score normalisation. For each equalised volume, the mean and standard deviation are computed only from voxels belonging to the brain mask, excluding background voxels from the estimation of the intensity distribution. The complete volume is then normalised by subtracting this foreground mean and dividing by the foreground standard deviation, producing brain voxels with approximately zero mean and unit variance. After normalisation, voxels outside the mask are set to zero so that non-brain regions do not contribute information to the subsequent models.

**Figure 2:**
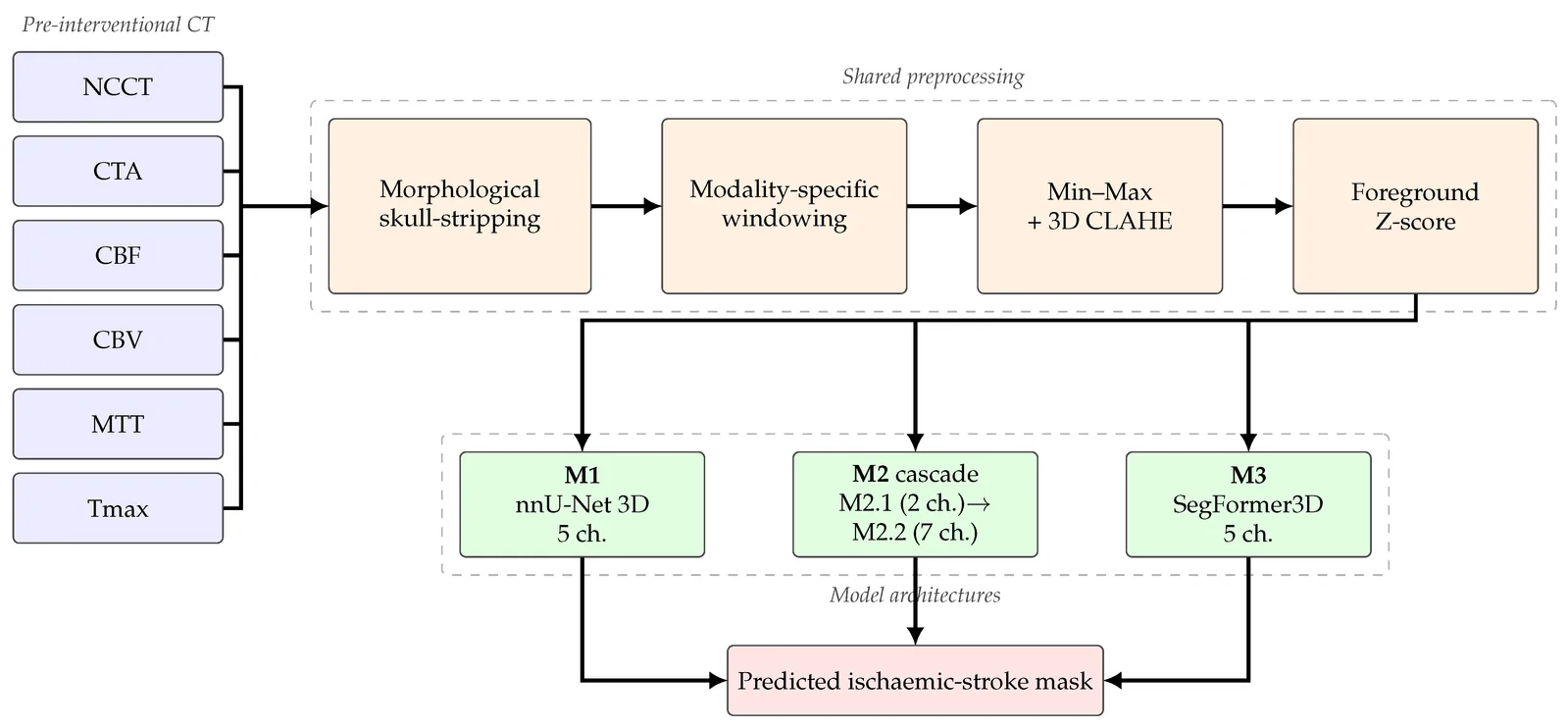
End-to-end pipeline used for the controlled comparison. The six pre-interventional CT-derived modalities (NCCT, CTA, CBF, CBV, MTT, and Tmax) are first processed with the same preprocessing chain for all patients and models: morphological skull-stripping removes non-brain tissue, modality-specific windowing clips intensities to clinically meaningful ranges, min–max normalisation rescales each channel, 3D CLAHE enhances local contrast, and foreground Z-score normalisation standardises the brain region while suppressing the background. The resulting preprocessed volumes are then provided to three segmentation strategies: M1, a single-stage 5-channel nnU-Net; M2, a two-stage nnU-Net cascade in which a 2-channel first-stage prediction is injected as an additional prior into a 7-channel second stage; and M3, a 5-channel SegFormer3D model. All branches finally produce a predicted ischaemic-stroke mask.

Finally, spatial resampling is applied. For M1 and M2, the nnU-Net planner [5] automatically rescales the volumes to the median spacing of the working set, which is [2.0;0.41;0.41] mm (anisotropic, Z:XY aspect ratio ≈5:1). The planner detects this anisotropy and configures the encoder kernels and strides accordingly. For M3, no spacing transform is applied; the network operates directly on the native voxel grid. The complete workflow, from multimodal input selection to shared preprocessing and model-specific segmentation strategies, is summarised in Figure 2.

### 3.4. Model Architectures

Three deep learning strategies are compared. M1 and M2 use the nnU-Net v2 framework [5] in its 3D full-resolution configuration, and M3 employs SegFormer3D [28], a hierarchical Transformer architecture.

#### 3.4.1. M1 (Single-Stage nnU-Net)

M1 is a 3D nnU-Net trained with 5 input channels (CTA, CBF, CBV, MTT, Tmax), replicating the input scheme of the challenge-winning team Kurtlab [8]. The architecture is the PlainConvUNet variant of 3d_fullres, automatically configured by the planner from the dataset fingerprint: 6 stages, feature widths [32,64,128,256,320,320], instance normalisation and Leaky ReLU activation. The first two stages use 1×3×3 kernels and 1×2×2 strides, deferring downsampling on the axial axis until the in-plane resolution is reduced to a comparable scale, in response to the severe anisotropy of the dataset. Kurtlab used the ResEnc L variant with 10-fold cross-validation; M1 uses PlainConvUNet on a single fold due to the compute limitations of Google Colab (Section 3.6). The resulting nnU-Net backbone configuration shared by M1 and the two M2 stages is summarised in Table 3.

#### 3.4.2. M2 (Two-Stage Cascade)

**Figure 3:**
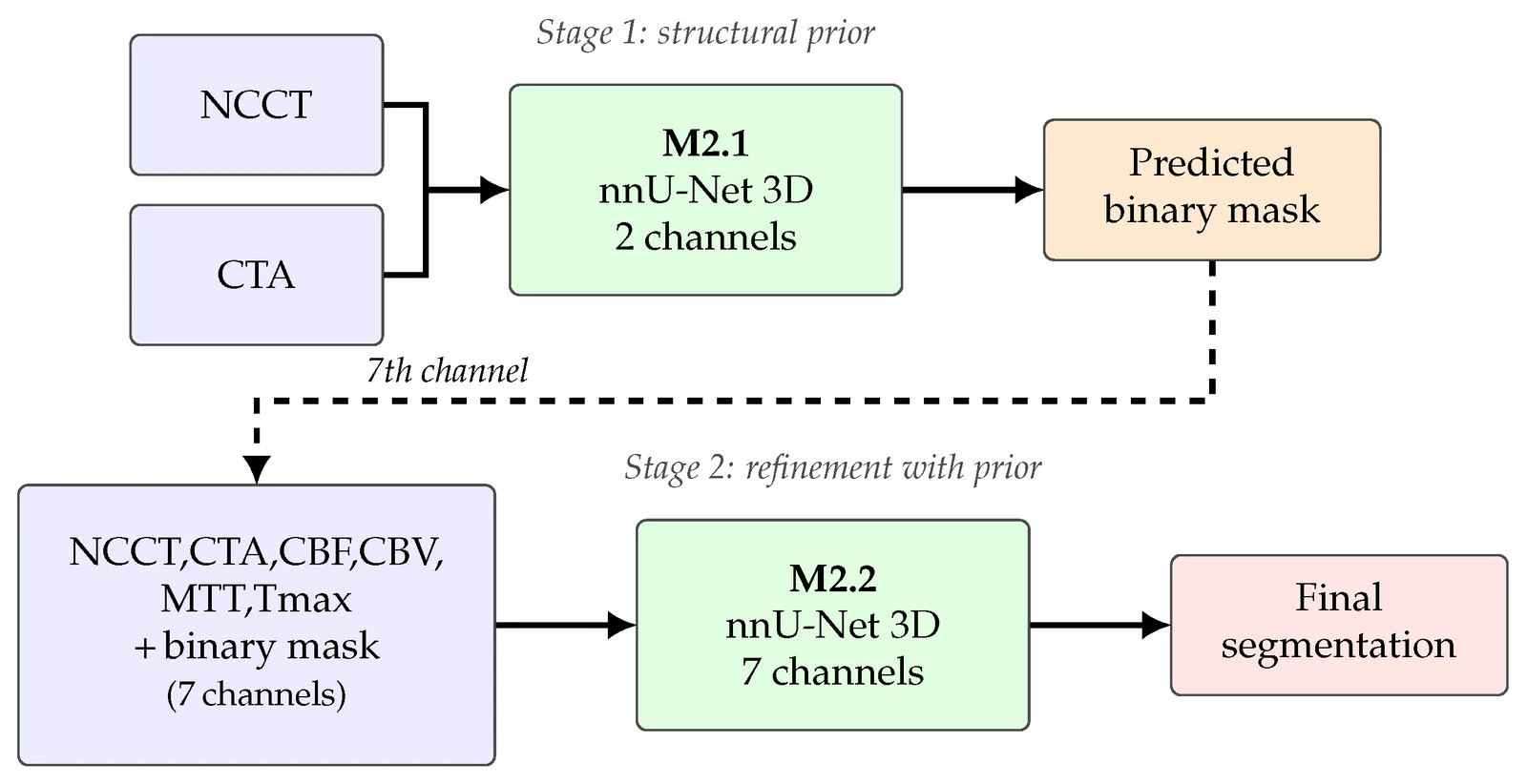
Two-stage cascade (M2). M2.1 trains on NCCT and CTA to produce a binary lesion prior; M2.2 trains on all six modalities concatenated with that prior. At inference time, the two stages run sequentially.

M2 combines the previous preprocessing pipeline with the multi-channel fusion scheme of the second-ranked team AMC-Axolotls [7]. The first stage (M2.1) is a 3D nnU-Net trained with 2 channels (NCCT and CTA) that generates a preliminary lesion mask. The second stage (M2.2) receives 7 channels (NCCT, CTA, CBF, CBV, MTT, Tmax, plus the binary mask of M2.1) and produces the final segmentation. Both stages share architecture, framework, and training configuration with M1. The 7th channel mask was generated by the already trained M2.1 using nnUNetv2_predict on the full pool of 133 patients, so the injected predictions are “in-fold” (optimistic), not out-of-fold. The two-stage information flow of the cascade, including the use of the first-stage mask as an additional input channel, is illustrated in Figure 3. This methodological detail is examined in Section 5.

#### 3.4.3. M3 (SegFormer3D)

**Figure 4:**
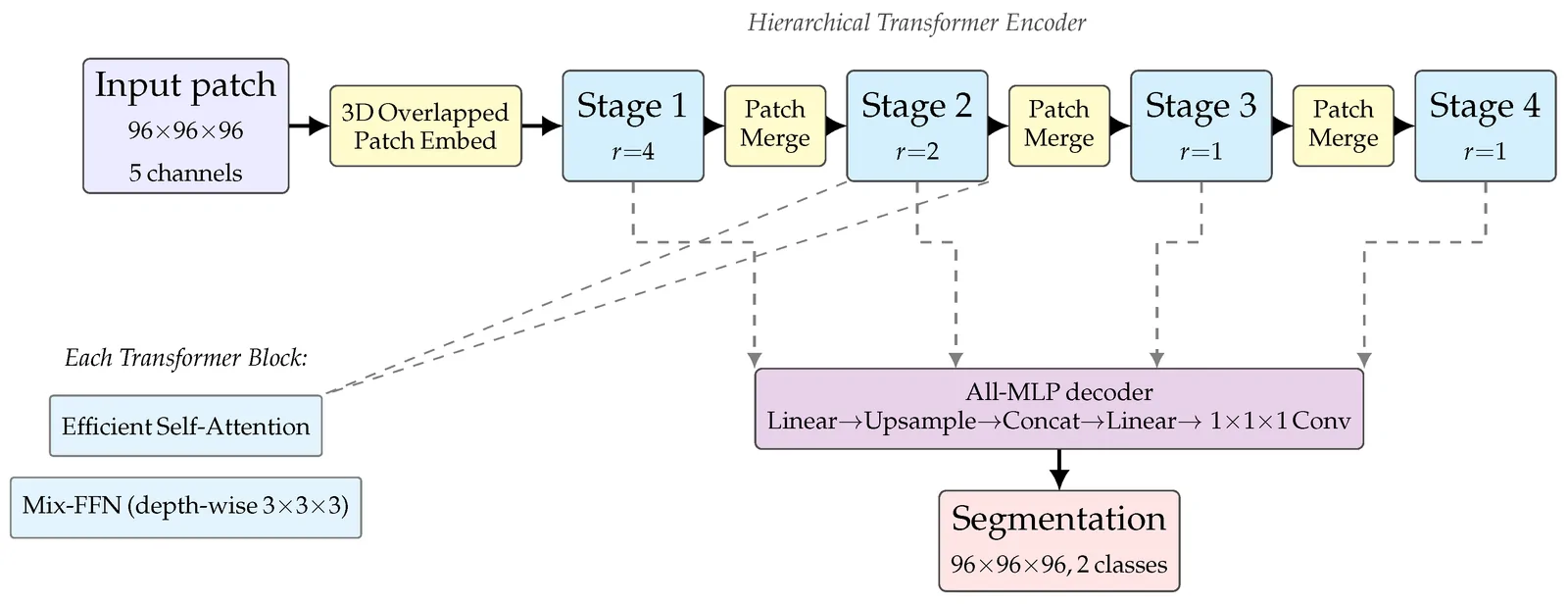
SegFormer3D architecture (M3). A four-stage hierarchical Transformer encoder processes $96^{3}$-voxel patches; each stage applies Efficient Self-Attention (reduction factors r=4,2,1,1) and Mix-FFN blocks. The all-MLP decoder aggregates multi-scale features into the final two-class segmentation. Adapted from [28].

M3 is the SegFormer3D architecture by Perera et al. [28], using the public implementation from the OSU-PCV Lab and the default configuration (in_channels = 5, num_classes = 2). With 4.5 M parameters and 17.5 GFLOPS, it is more than an order of magnitude smaller than nnFormer (150.5 M), UNETR (92.5 M), or SwinUNETR (62.8 M). The hierarchical encoder processes 96×96×96-voxel patches in four stages, applying in each one an Efficient Self-Attention module with reduction factors r=4,2,1,1 and a Mix-FFN module that combines a 3×3×3 depth-wise convolution with a feed-forward network. The Mix-FFN injects implicit positional information, eliminating the fixed input-size restriction that makes nnFormer incompatible with the heterogeneous volumes of ISLES’24. The all-MLP decoder projects the four multi-scale feature maps to a common dimension, upsamples, concatenates, and fuses them via a final linear projection. Given the dataset’s median spacing [2.0;0.41;0.41] mm, the 96-voxel patch covers ≈192 mm in Z (the full cranial height) and only ≈39 mm in-plane, compared with the 80 mm and 92 mm of the nnU-Net patch. The implications of this asymmetric field of view are analysed in the discussion. The main encoder–decoder components of the SegFormer3D implementation used for M3 are shown in Figure 4.

### 3.5. Training

Regarding the loss function, all three models are trained with a 1:1 weighted combination of soft Dice loss and voxel-wise cross-entropy. For nnU-Net, it is the default loss of the framework [5]; for SegFormer3D, MONAI’s DiceCELoss [38] is used with one-hot encoding of the target and softmax activation at the output.

Regarding the models trained with nnU-Net (M1 and the two cascade stages M2.1 and M2.2), fold 0 was used with the SGD optimiser with Nesterov momentum 0.99 and initial learning rate 0.01, reduced by a PolyLR scheduler with exponent 0.9. The single-stage model M1 and the second cascade stage M2.2, which are the networks evaluated as final outputs, were trained for 250 epochs; the intermediate first-stage model M2.1, which only produces the binary prior injected into M2.2, was trained for 300 epochs. Each epoch processes 250 mini-batches of 40×224×192-voxel patches sampled with foreground oversampling (probability 0.33). Regarding data augmentation, the default nnU-Net pipeline was used [5], which consists of 3D rotations, scaling [0.7–1.4], elastic deformations, additive Gaussian noise, multiplicative brightness scaling, contrast data augmentation by gamma transformation, low-resolution simulation and axial mirroring.

On the other hand, M3 (SegFormer3D) was also trained for 250 epochs using fold 0, the AdamW optimiser [43], an initial learning rate of $1\times 10^{-4}$, a weight decay of $10^{-5}$, and the same PolyLR scheduler. Its batch size was 2 for training and 1 for validation, with validation performed at each epoch using a sliding-window inferer with 96×96×96 patches and an overlap of 0.5; the checkpoint with the highest validation Dice was kept. Regarding data augmentation, the minimal MONAI chain was used [38]: NormalizeIntensityd, SpatialPadd to 96×96×96, RandCropByPosNegLabeld (positive:negative ratio 1:1, 2 samples per volume) and RandFlipd (probability 0.5, axis 0). This asymmetry in data augmentation is discussed later in depth due to the confound it produces in the comparison with M1 and M2. The final training configuration for the nnU-Net stages and SegFormer3D is summarised in Table 4.

**Figure 5:**
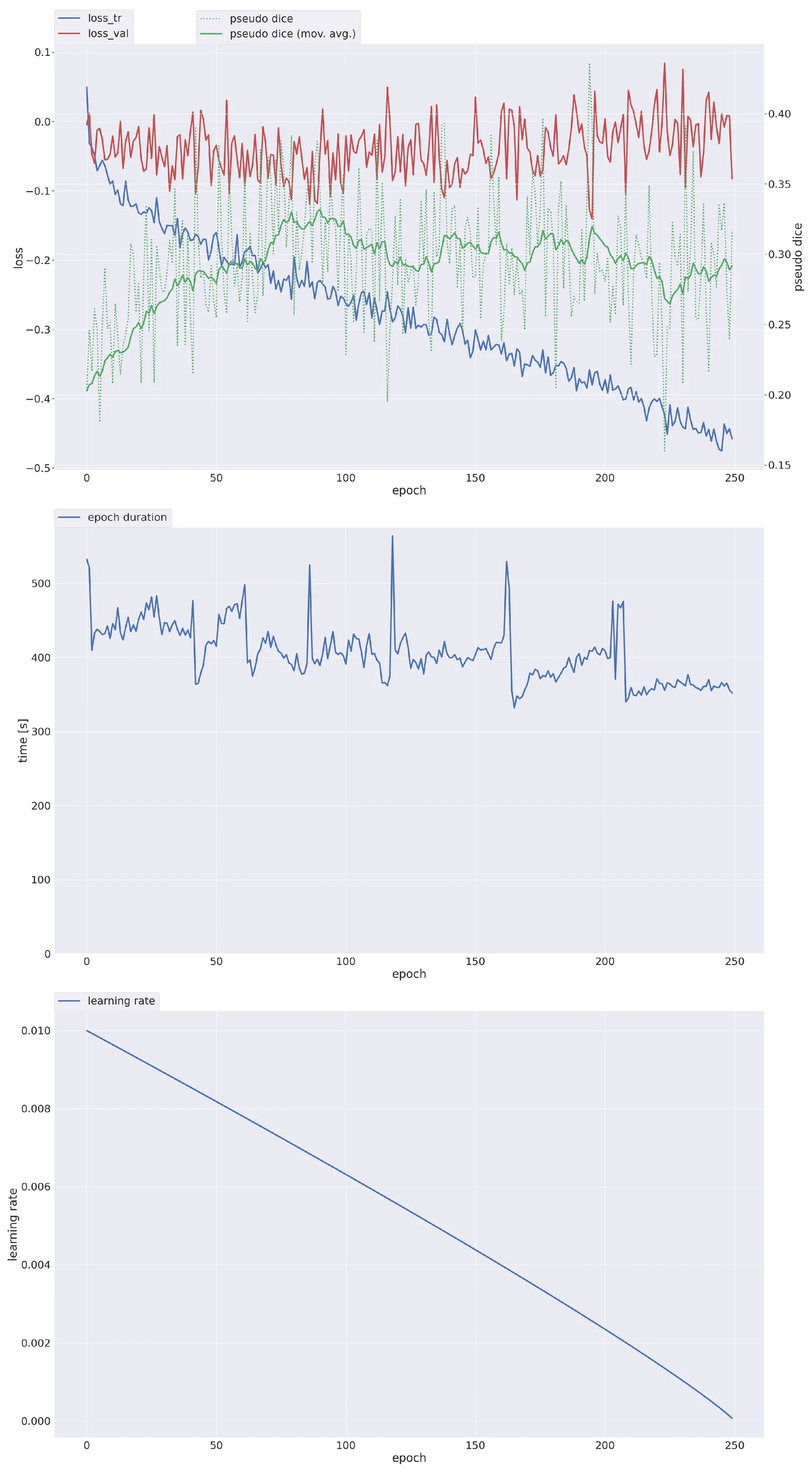
Training curves of M1 (single-stage nnU-Net 3D, 5 channels) over 250 epochs. The panel shows the training loss and the validation loss (left axis) together with the validation pseudo-Dice (right axis), an online approximation of the Dice computed on the validation patches. The nnU-Net loss is negative by construction, as it combines the Dice coefficient (with negative sign) with cross-entropy, so a lower value indicates a better fit. The training loss decreases steadily towards the range −0.4 to −0.5 while the validation loss remains noisy and essentially stable (between −0.1 and 0.1); the validation pseudo-Dice rises from about 0.20 to a peak of 0.30–0.35 and settles around 0.30. The best validation checkpoint was obtained at epoch 90. The figure also reports the per-epoch duration and the PolyLR learning-rate decay; the isolated peaks in the duration curve correspond to fluctuations of the shared Colab environment and not to changes in the model.

**Figure 6:**
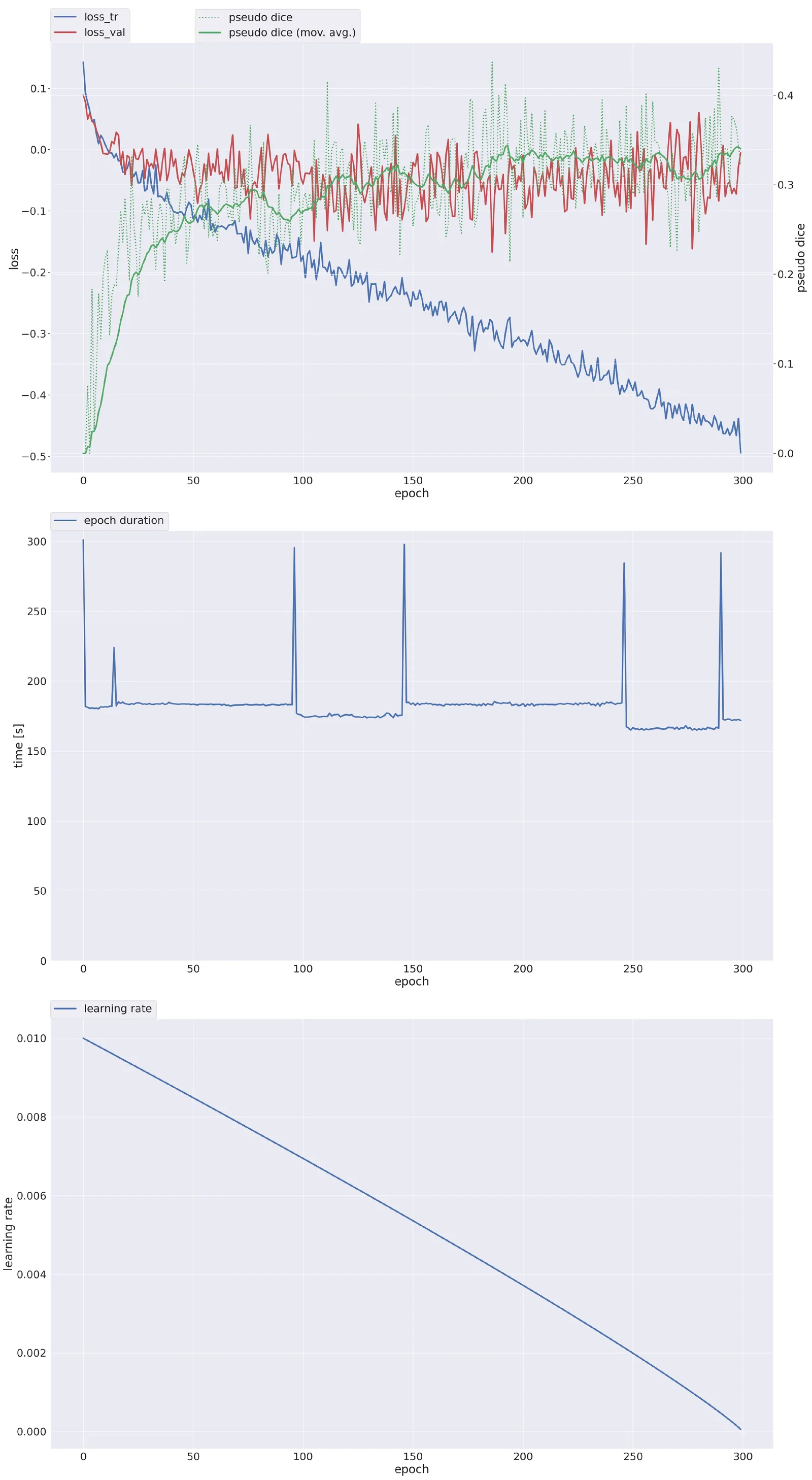
Training curves of M2.1 (first cascade stage, nnU-Net 3D, 2 channels: NCCT and CTA) over 300 epochs, with the same axes as Figure 5 and, as for M1, the per-epoch duration and the PolyLR decay. M2.1 is trained without any injected prior, so it behaves as an ordinary single-stage network: the training loss decreases steadily towards the range −0.4 to −0.5 while the validation loss stays noisy and essentially stable, and the validation pseudo-Dice rises to a plateau around 0.30, closely matching the convergence of M1 (Figure 5). This stage produces the binary prior that is injected as the seventh channel of M2.2; its similarity to M1, contrasted with the earlier saturation of M2.2, illustrates the in-fold effect discussed in Section 5.2.

**Figure 7:**
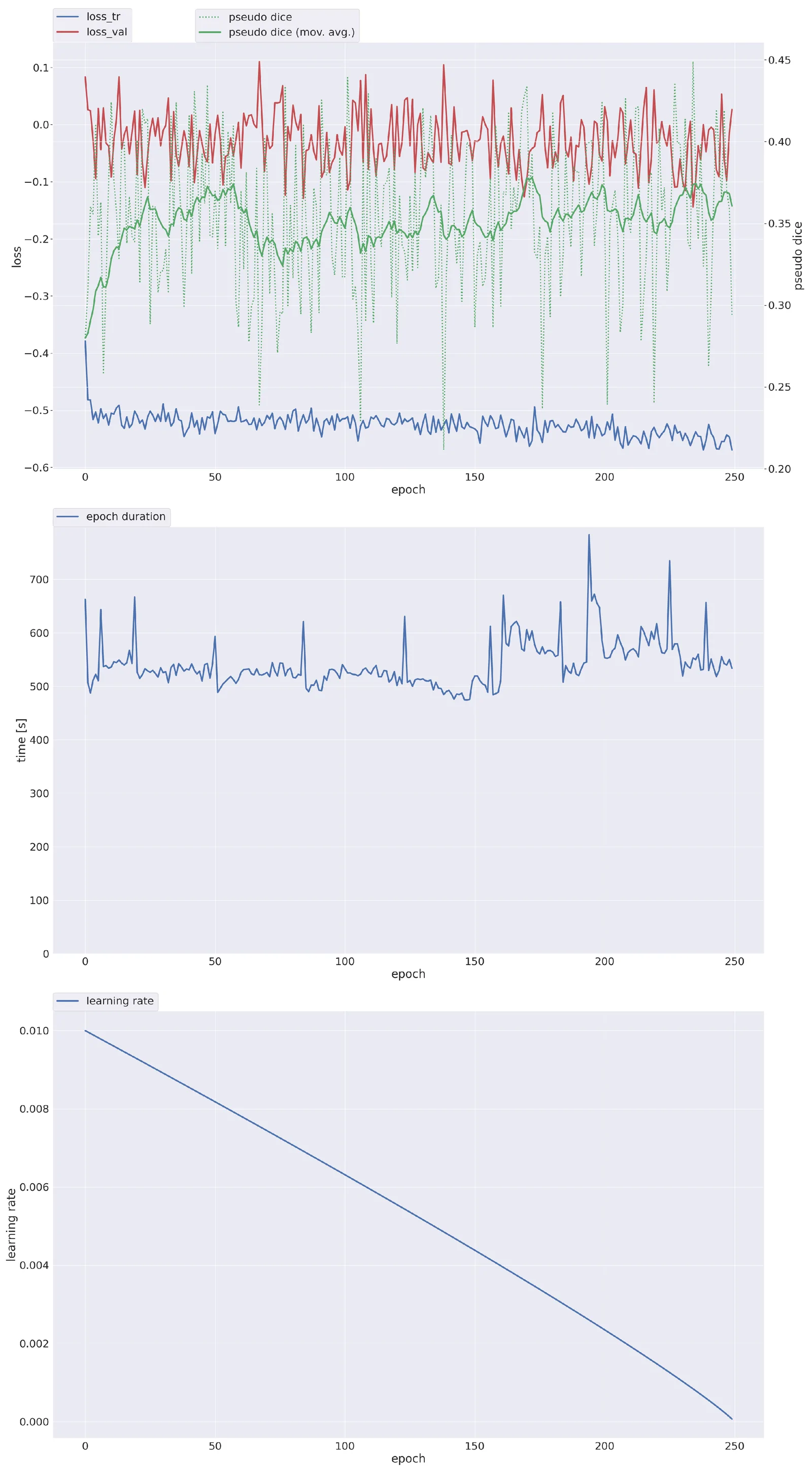
Training curves of M2.2 (second cascade stage, nnU-Net 3D, 7 channels) over 250 epochs, with the same axes as Figure 5 and, as for the other nnU-Net models, the per-epoch duration and the PolyLR decay. Compared with M1 (Figure 5) and with the first stage M2.1 (Figure 6), the training loss decreases faster and reaches its plateau (approximately −0.5 to −0.55) earlier, and the validation pseudo-Dice rises and stabilises earlier as well, settling around 0.35–0.37. This earlier saturation could be attributed to the in-fold generation of the seventh-channel prior discussed in Section 5.2: because the prior is produced by M2.1 on patients it had already seen during training, the second stage receives an optimistic input that it can fit sooner. The best validation checkpoint was obtained at epoch 172.

**Figure 8:**
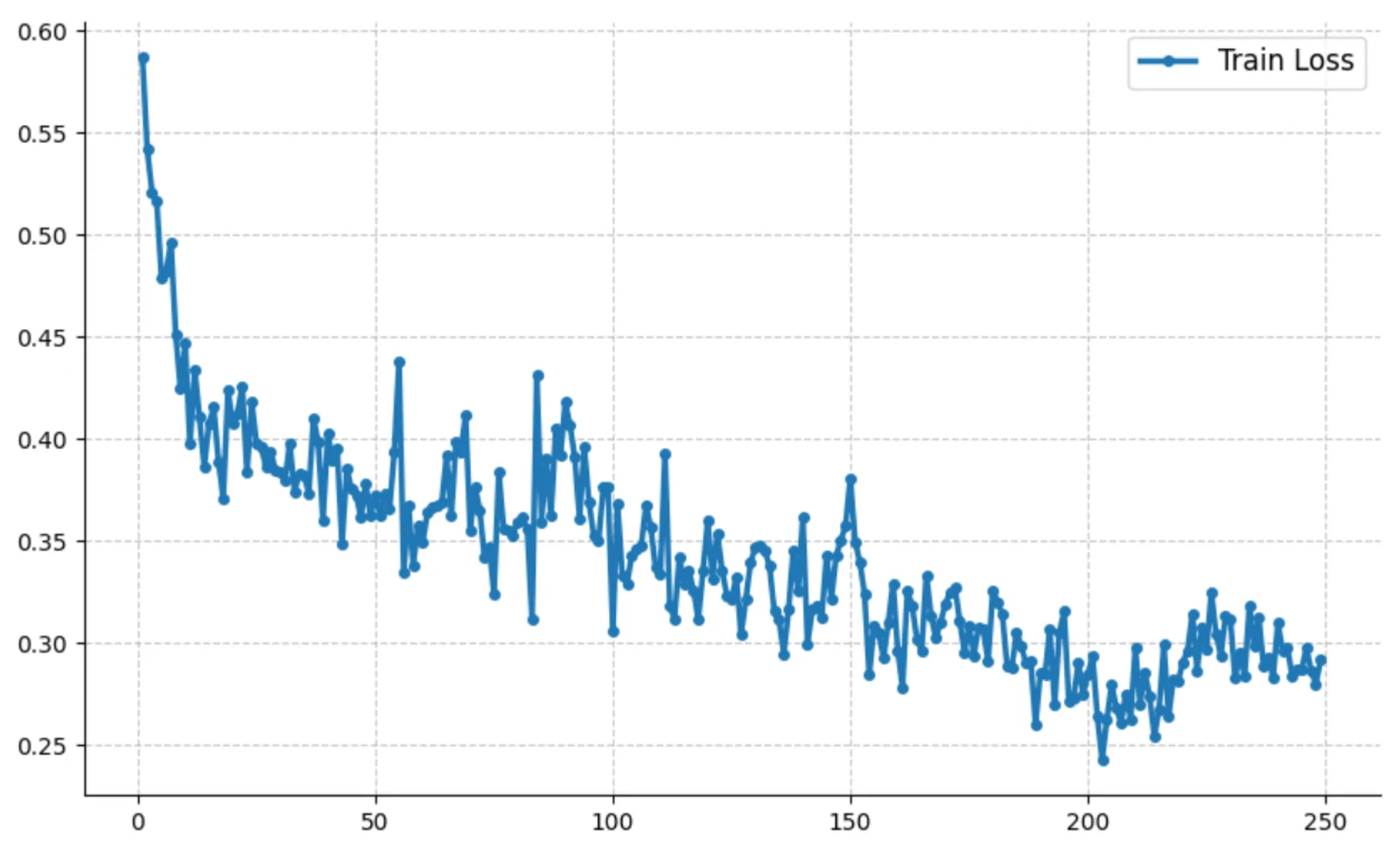
Training curve of M3 (SegFormer3D) over 250 epochs. Only the training loss recorded during fitting on Google Colab is available for this model; the MONAI DiceCELoss is positive, so a lower value indicates a better fit. The loss decreases from about 0.55–0.60 and stabilises around 0.28–0.30 more gradually and without a clear plateau, which suggests the model could keep improving with more epochs or more data. The absence of an equivalent validation curve, a limitation of the record available for this model, prevents confirming its generalisation behaviour. The best validation checkpoint was saved at epoch 167.

The training dynamics of the four trained networks are shown in Figure 5, Figure 6, Figure 7 and Figure 8. For the nnU-Net models (M1, M2.1, and M2.2), the framework logs the training and validation loss together with the validation pseudo-Dice, an online approximation of the Dice computed on the validation patches; for M3 (SegFormer3D), only the training loss recorded during fitting on Google Colab is available. The nnU-Net loss takes negative values because it combines the Dice coefficient (with negative sign) with cross-entropy, so a lower value indicates a better fit. In M1, the training loss decreases steadily while the validation loss remains noisy and essentially flat, and the validation pseudo-Dice rises to a plateau around 0.30, with the best validation checkpoint at epoch 90. The first cascade stage M2.1, trained on 2 channels (NCCT and CTA) for 300 epochs, follows a very similar trajectory to M1, with the validation pseudo-Dice reaching a comparable plateau around 0.30: this is expected, as M2.1 is trained without any injected prior and therefore behaves like an ordinary single-stage network. The second cascade stage M2.2, in contrast, reaches its plateau earlier and slightly higher, with the training loss and the validation pseudo-Dice (0.35–0.37) saturating sooner than in both M1 and M2.1. This earlier saturation could be attributed to the in-fold generation of the seventh-channel prior (Section 5.2): since M2.1 produced that prior on patients it had already seen during training, M2.2 receives an optimistic input that it can fit more quickly. The contrast between the M1-like convergence of M2.1 and the earlier saturation of M2.2 provides a visual illustration of this in-fold effect. The M3 training loss decreases more gradually towards 0.28–0.30 without a clear plateau, and its best validation checkpoint was kept at epoch 167. The growing gap between a still-decreasing training loss and a stagnant validation curve is a mild sign of overfitting, consistent with the limited training set of 106 patients and with the final performance observed on the test set.

### 3.6. Experimental Environment

It is worth noting that for the use of the dataset in Google Colaboratory, the initial size of the dataset had to be reduced, from approximately 100 GB, to 7–15 GB depending on the model. This was done by eliminating the entire raw branch except for NCCT, because the NCCT file was not included in the derivatives branch; in that branch, all images are spatially aligned with NCCT, which was not duplicated. The phenotype branch was also eliminated, as it was not used by most teams, and its influence on model prediction was not demonstrated. Regarding the follow-up session, only the ground truth was kept from the derivatives branch, and regarding the admission images, the CTA and the four perfusion maps derived from the CTP were kept. The cow-msk and lvo-msk were eliminated, as no team used them, and the CTP was eliminated due to its large size and because only a minority of the teams that participated in the challenge used it.

All training and inference were performed on the Google Colab free tier, with an NVIDIA Tesla T4 GPU in each run. Persistent storage was managed through Google Drive, copying the preprocessed volumes to the local Colab instance at the start of each session. All training pipelines implemented per-epoch checkpointing to allow resumption after an environment disconnection because of the usage limits of the free version. The software environment used Python 3.12, PyTorch 2.8.0, nnU-Net v2 2.7.0 [5], MONAI 1.5.1 [38], scikit-image 0.26.0, SciPy 1.16.0, nibabel 5.4.2, SimpleITK 2.5.2, and cc3d 3.24.0 [42]. Because the Google Colab environment was not version-pinned, these versions correspond to the Colab-preinstalled defaults (for Python, PyTorch and SciPy) or to the latest stable releases available on PyPI at the time of execution (March 2026).

### 3.7. Evaluation Metrics

All models were evaluated using the official ISLES’24 metrics [7], computed from the predictions obtained for the 15 patients reserved as the held-out test set.

DSC (*Dice Similarity Coefficient*) quantifies the volumetric overlap between the predicted and reference masks. It ranges from 0 to 1, with higher values indicating better agreement, and is used as the primary segmentation metric. AVD (*Absolute Volume Difference*, in mL) measures the absolute difference between the predicted and reference lesion volumes. This metric assesses volumetric accuracy independently of the spatial localisation of the predicted mask. Lesion-wise F1 evaluates detection performance at the lesion-instance level. A predicted lesion is considered a true positive only when its IoU with a reference lesion is at least 20%; greedy one-to-one matching is then applied, thereby penalising missed lesions, false positives, and lesion fragmentation. ALCD (*Absolute Lesion Count Difference*) measures the absolute difference between the number of connected components in the prediction and in the reference mask. It complements lesion-wise F1 by quantifying over-segmentation and under-segmentation in terms of lesion count. Connected components were computed in 3D using 26-neighbour connectivity with cc3d [42].

## 4. Results

This section presents the performance results of each of the trained models when evaluated on the held-out test set of 15 patients. The presentation of these results is organised into four sections: overall performance per model (Section 4.1), controlled pairwise comparisons that isolate individual experimental factors (Section 4.2), contextual comparison with the official ISLES’24 leaderboard (Section 4.4), and finally, a qualitative inspection of representative cases (Section 4.5).

### 4.1. Overall Performance on the Held-Out Test Set

Table 5 summarises the four official ISLES’24 metrics (DSC, AVD, lesion-wise F1 and ALCD) for the four trained models on the same 15-patient test set. Values are reported as mean (standard deviation) across patients.

The two cascade stages (M2.1 and M2.2) attained the highest DSC, both around 0.224. M1 followed at 0.210, and M3 reached 0.163. AVD was minimum for M1 (23.70 mL) and stayed between 27.28 and 28.38 mL for the remaining three models. Lesion-level F1 was maximum in M1 (7.60%), with M2.2 second (5.30%), M2.1 third (4.60%) and M3 last (2.30%). The ALCD ordering placed both cascade stages first (5.40), followed by M1 (6.20) and M3 (7.27). The DSC standard deviations ranged between 0.237 (M1) and 0.178 (M3), the latter being the highest inter-patient consistency among the four models.

### 4.2. Controlled Pairwise Comparisons

The shared preprocessing pipeline, the identical patient partition and the equivalent training schedule allow direct pairwise comparisons in which only one experimental factor varies between the two models being compared. Table 6 reports three of these comparisons: the architectural comparison (M3 vs. M1), the effect of injecting the prior inside the cascade (M2.1 vs. M2.2), and the comparison between the single-stage approach and the full cascade (M1 vs. M2.2).

The M3 vs. M1 comparison isolates the effect of the architecture given that the input modalities, the preprocessing, the training fold and the validation protocol are identical. M1 outperformed M3 on the four metrics, with a DSC difference of 0.047, an AVD difference of 3.58 mL, an F1 difference of 5.30 percentage points, and an ALCD difference of 1.07. The four metrics agreed in direction.

The M2.1 vs. M2.2 comparison isolates the effect of injecting the binary prediction of M2.1 as the seventh input channel. The DSC difference was 0.001 in favour of M2.2, the AVD increased by 0.69 mL, the F1 increased by 0.70 percentage points, and the ALCD remained identical at 5.40. None of the four metrics moved by a magnitude comparable to its own inter-patient standard deviation.

The M1 vs. M2.2 comparison isolates the strategic decision between a single-stage 5-channel network and the full two-stage 7-channel cascade, with the same backbone architecture at both ends. M2.2 obtained a DSC 0.014 above that of M1, an AVD 4.68 mL above, an F1 2.30 percentage points below, and an ALCD 0.80 below. The four metrics did not agree in direction.

### 4.3. Statistical Analysis of the Paired Comparisons

**Figure 9:**
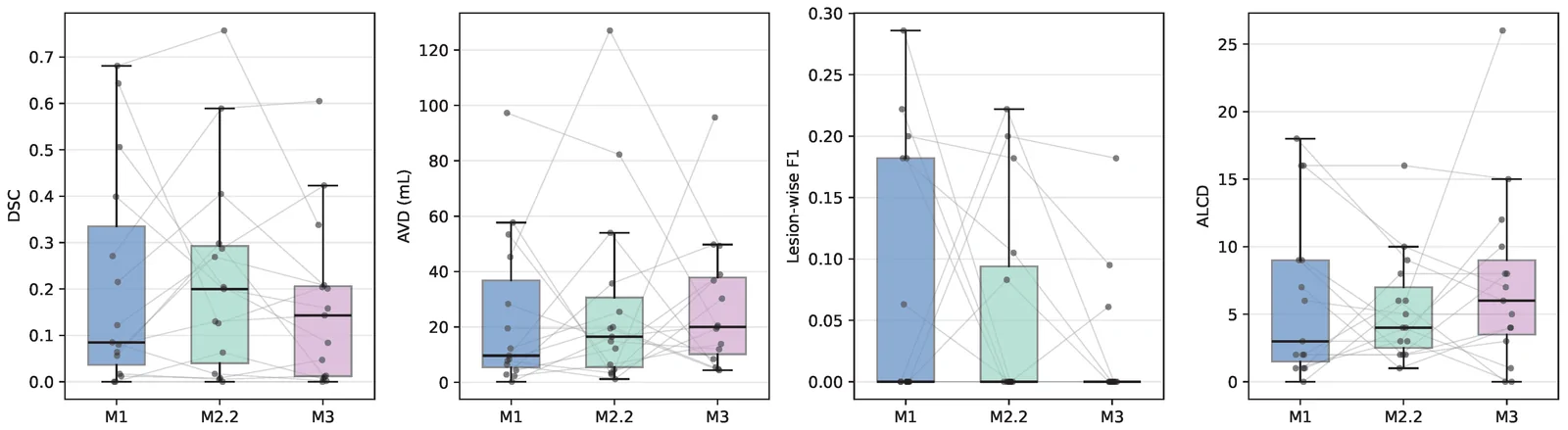
Per-patient distribution of the four official ISLES’24 metrics across the 15 held-out test patients for M1, M2.2, and M3. From left to right, the panels show the Dice similarity coefficient (DSC, higher is better), the absolute volume difference (AVD, in mL, lower is better), the lesion-wise F1-score (higher is better) and the absolute lesion count difference (ALCD, lower is better). In each panel, the three boxes correspond, from left to right, to M1, M2.2, and M3. Each box shows the median and the interquartile range; whiskers extend to 1.5 × IQR, and individual patients are overlaid as black points, with grey lines connecting the same patient across the three models to make the paired structure explicit. The large overlap between the distributions, and the crossing of the per-patient lines, are consistent with the absence of statistically significant differences reported in Table 8.

Given the small evaluation cohort (15 patients) and the fact that the reported standard deviations are comparable to the mean values, the paired differences of Section 4.2 were assessed with a formal statistical analysis rather than by point estimates alone. For each metric and each model pair, the per-patient paired difference was computed over the 15 test patients. A two-sided Wilcoxon signed-rank test (exact, no continuity correction) was used as a non-parametric paired test, and a 95% confidence interval (CI) for the mean paired difference was obtained by paired bootstrap resampling (10,000 resamples, seed 42). Because four metrics are reported, the DSC (the primary metric) is additionally corrected across the three model pairs with the Holm procedure. Median and interquartile range (IQR) are reported in Table 7 alongside the mean and standard deviation of Table 5. The distribution of per-patient metrics is shown in Figure 9.

Across all three model pairs and the four metrics, no paired comparison reached statistical significance at the conventional α=0.05 level, and every bootstrap confidence interval for the mean difference included zero (Table 8). The smallest uncorrected *p*-values corresponded to the lesion-wise F1 advantage of M1 over M3 (p=0.109) and of M2.2 over M3 (p=0.094), both above the significance threshold; after Holm correction of the primary DSC metric, no comparison remained close to significance. The nominal DSC advantage of the cascade over the single-stage network (Δ=0.014) and the near-tie inside the cascade (Δ=0.001) are therefore not statistically distinguishable on this cohort. These results indicate that, with 15 patients, the study is not powered to establish that any single model genuinely outperforms another, and the orderings observed in Table 5 should be read as descriptive of this dataset rather than as evidence of a systematic ranking. This limited statistical power is discussed further in Section 5.5.

### 4.4. Comparison with ISLES’24 Challenge Submissions

Table 9 places the four trained models alongside the five reference entries from the ISLES’24 leaderboard most relevant for this comparison. We can see the three top-ranked submissions (Kurtlab, AMC-Axolotls, Ninjas) [7,8], and the two best Transformer-based entries (MIPLAB-PrediCTP, hybrid 2D CNN-Transformer; QuiiL/MoReT, pure 3D Transformer) [7,36]. The two evaluation regimes are not directly equivalent given that, beyond the test set size (15 internal vs. 100 hidden), the reference submissions used larger training pools (typically 150 cases under multi-fold cross-validation, with submission of the best-performing fold) more capable nnU-Net variants in two of the three top teams (large or medium residual encoder instead of PlainConvUNet), and SynthStrip-based skull-stripping [35]. The models trained in this work used a single fold over a 133-patient pool with morphological skull-stripping (Section 3.3). Because the two regimes differ simultaneously in training-pool size, cross-validation strategy, backbone variant, preprocessing and test-cohort size, the entries in Table 9 are *not* directly comparable and must not be read as a head-to-head benchmark. They are provided solely as a qualitative contextual reference to situate the magnitude of the present results within the published challenge landscape, and no claim of superiority or inferiority against the official submissions is made on the basis of these numbers.

On the single-stage CNN axis (M1 vs. Kurtlab), DSC differed by 0.075 (0.210 vs. 0.285) and AVD by 2.47 mL (23.70 vs. 21.23). F1 differed by 6.80 percentage points (7.60% vs. 14.40%), and ALCD by 0.98 (6.20 vs. 7.18, with M1 lower). Beyond the training-pool and test-set differences common to all comparisons in this section, the architectural variants are not equivalent: Kurtlab used the large residual encoder of nnU-Net under 10-fold cross-validation [8], while M1 used the PlainConvUNet variant trained on a single fold.

On the cascade axis (M2.2 vs. AMC-Axolotls), the DSC of M2.2 stayed below that of AMC-Axolotls by 0.039 (0.224 vs. 0.263), AVD differed by 7.07 mL (28.38 vs. 21.31), F1 by 9.64 percentage points (5.30% vs. 14.94%), and ALCD by 2.26 (5.40 vs. 7.66, with M2.2 lower). Both pipelines share the cascaded nnU-Net 3d_fullres backbone; the principal methodological gap is the training pool and the cross-validation regime.

On the Transformer axis (M3 vs. MIPLAB-PrediCTP and QuiiL/MoReT), M3 obtained the highest DSC of the three, 0.163, against 0.139 of MIPLAB-PrediCTP and 0.068 of QuiiL/MoReT. The AVD of M3 (27.28 mL) stayed 50.10 mL below that of MIPLAB-PrediCTP (77.38 mL) and 140.78 mL below that of QuiiL/MoReT (168.06 mL). F1 was 2.30% for M3, against 1.79% and 1.12%, respectively. ALCD was 7.27 for M3, 20.02 for MIPLAB-PrediCTP, and 7.11 for QuiiL/MoReT, the latter marginally below M3. In this contextual sense, M3 reported higher DSC, lower AVD and higher F1 than the two Transformer-based entries of the official leaderboard, despite the smaller training pool and the single-fold regime; given the methodological asymmetry described above, this observation is offered only as qualitative context and not as a controlled comparison.

### 4.5. Qualitative Analysis

**Figure 10:**
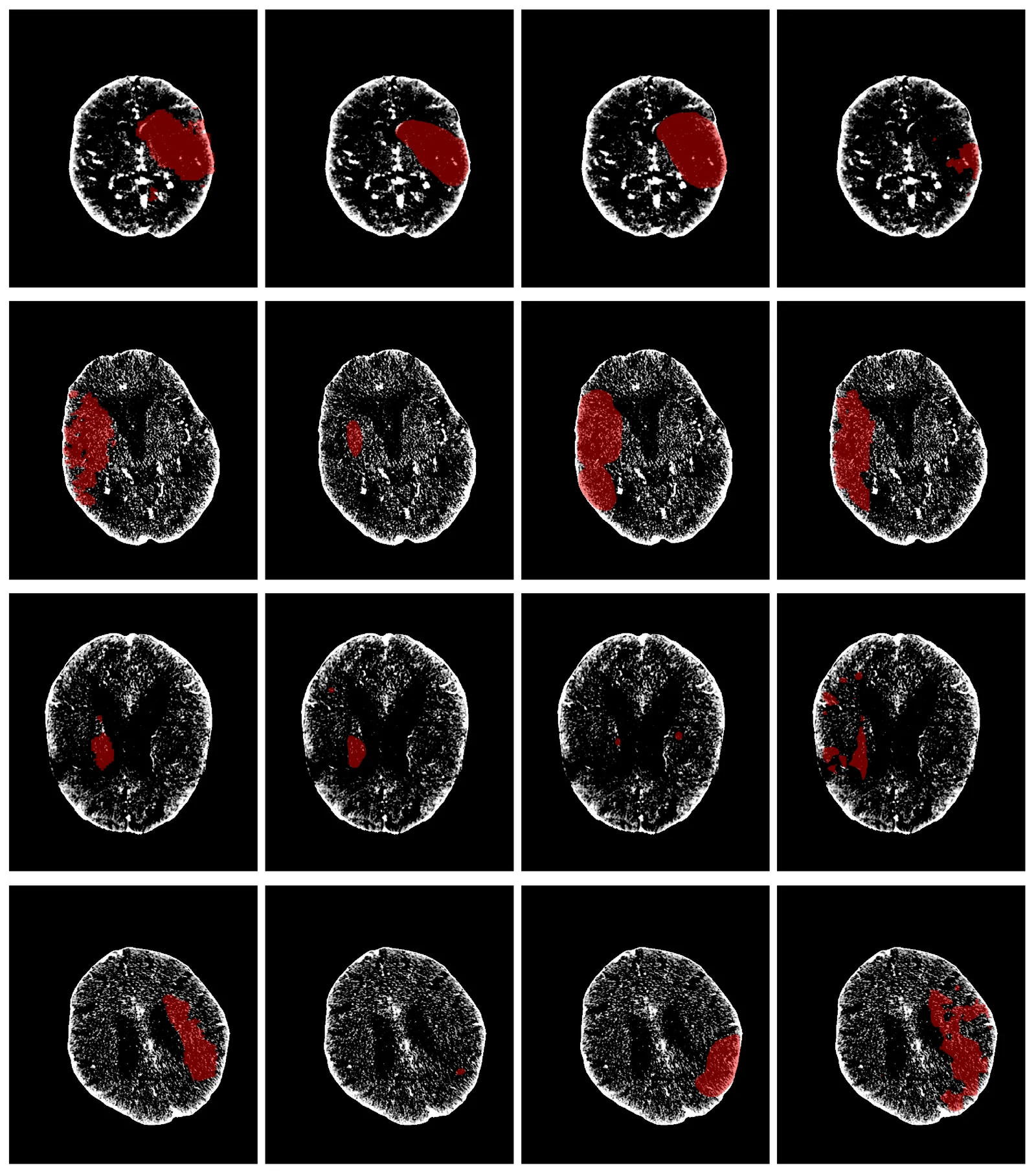
Qualitative comparison of the three segmentation strategies on four representative cases from the held-out test set. The figure is arranged in four rows and four columns. Columns, from left to right: ground-truth infarct mask, M1 prediction, M2.2 prediction, and M3 prediction, all overlaid on the CTA channel as anatomical reference (red region: lesion mask). Row 1 (sub-stroke0027): best CNN case; Transformer underperforms. Row 2 (sub-stroke0082): best Transformer case across the test set. Row 3 (sub-stroke0148): M1 success with simultaneous cascade failure. Row 4 (sub-stroke0007): severe fragmentation across all models.

Figure 10 shows representative cases from the test set for visual inspection. Each row corresponds to one patient and presents, on the same axial slice, the ground-truth infarct mask and the binary predictions of M1, M2.2 and M3, all overlaid on the CTA channel as an anatomical reference. The selected cases include both successful segmentations and characteristic failure patterns (volumetric over- and under-segmentation, lesion fragmentation, and isolated false-positive components). The corresponding per-patient quantitative metrics for these representative examples are reported in Table 10.

## 5. Discussion

The results of this study highlight four main points. First, preprocessing—especially modality-specific clinical windowing—appears to be a major determinant of performance in ISLES’24, influencing volumetric calibration and partly explaining the gap between controlled internal experiments and the official leaderboard. Second, the proposed two-stage cascade provides only marginal improvements over the single-stage nnU-Net despite increasing input dimensionality and computational cost, suggesting that the additional binary prior contributes limited information beyond the perfusion channels. Third, under identical preprocessing and data partitions, the CNN-based models showed higher point estimates than the Transformer model, but paired testing found these differences to be non-significant on the 15-patient cohort, and patient-level results reveal complementary failure and success patterns that may motivate future ensemble strategies. Finally, all models remain limited by small-lesion detection, fragmentation, restricted test-set size, the single-seed training regime, and the predictive nature of the task, so the present findings should be interpreted as methodological evidence specific to this dataset and configuration rather than as a clinically deployable segmentation system.

### 5.1. Preprocessing as the Dominant Factor in the Challenge Gap

The 0.075 DSC gap between M1 (0.210) and the official Kurtlab entry (0.285) can be attributed to four differences. The first two are the training set size, which increased from 133 patients to 150, and the test set size, with 15 patients for M1 and 100 for Kurtlab, which makes the results obtained in Kurtlab’s evaluation statistically more robust. Likewise, Kurtlab used a 10-fold cross-validation regime, compared with M1, which used a single fold. Finally, the architectural variant of nnU-Net must be taken into account, which was PlainConvUNet for M1 and a large residual encoder for Kurtlab [8,37].

A more revealing comparison is AMC-Axolotls vs. Kurtlab on the official leaderboard, as both teams used the nnU-Net 3D full-resolution backbone, applied skull-stripping and trained with cross-validation on the full 150-patient pool, but AMC-Axolotls obtained a DSC of 0.263 against 0.285 [7]. The main difference between them was the windowing strategy. Kurtlab applied narrow modality-specific clinical windows (CBF [0–35], CBV [0–10], CTA [0–90] HU), whereas AMC-Axolotls used much wider clipping ranges (CBF [0–400], CBV [0–400], CTA [0–200] HU). This reinforces the idea that windowing acts as a clinical prior that concentrates the dynamic range on tissue values relevant to ischaemic core delineation [8]. M1 inherits this strategy and reaches an AVD of 23.70 mL, close to Kurtlab (21.23 mL) and below Ninjas (26.29 mL) despite the smaller training pool, suggesting that windowing-based preprocessing effectively calibrates the global lesion volume, while the residual DSC gap may be due to architectural differences and data volume.

A methodological caveat concerns the CLAHE step and its interaction with the perfusion-derived channels. The perfusion maps (CBF, CBV, MTT, Tmax) carry quantitative physiological meaning, and a contrast-enhancing operation could in principle distort the intensity values that the network reads as haemodynamic signal. In the present pipeline CLAHE is applied after modality-specific windowing and before foreground Z-score normalisation, and it operates as a local, order-preserving redistribution within the retained clinical range: because the equalisation is monotonic within each local neighbourhood, it widens the originally narrow intensity distribution of each channel to enhance local contrast without introducing values outside the normalised range or altering the relative ordering of tissue intensities, so the quantitative signal encoded by the perfusion maps is preserved rather than corrupted. We nonetheless acknowledge that a full ablation isolating the individual contribution of each preprocessing step (windowing and normalisation only; adding CLAHE; CLAHE applied before versus after normalisation) was not carried out for every model, mainly because of the training cost of repeating the full pipeline on the free-tier compute used in this work. The winning-team ablation reported by Ren et al. [8], which isolates the effect of windowing and of histogram equalisation on DSC, provides partial external support for the direction of these choices, but a dedicated component-wise ablation on the present dataset remains a clear line of future work.

### 5.2. Cascade Architecture: Marginal Gain and In-Fold Mask Generation

The two cascade stages produced almost indistinguishable metrics: ΔDSC of 0.001, ΔAVD of 0.69 mL, ΔF1 of 0.70 pp and ΔALCD of 0.00, all well below inter-patient standard deviations. This may have two explanations, the first being that the CTP-derived channels already contain the haemodynamic information of ischaemic tissue, so adding a binary mask derived only from NCCT and CTA does not provide additional information that the model cannot extract directly from the perfusion channels. Second, and more relevant from a methodological point of view, the masks injected as the seventh channel of M2.2 were generated by running M2.1 through the 133 patients, including the 27 on which it had been trained. In contrast, AMC-Axolotls avoided this problem by using five-fold cross-validation, generating predictions only for patients the model had not seen [7]. So, if the mask were truly helpful, this advantage should have translated into a visible improvement, not a difference of 0.001 in DSC. Moreover, the official ISLES’24 leaderboard reinforces the argument with examples such as team ufpr_ua, which obtained a DSC of 0.199 using exclusively CTA as input [7], surpassing several teams with more channels and indicating that selecting channels carefully matters more than adding more.

It must be emphasised that the in-fold generation of the prior biases this comparison *in favour* of the cascade, not against it: because M2.1 produced the seventh-channel mask on the full 133-patient pool, the second stage received, for 106 of those patients, lesion priors generated by a model that had already seen them during training. A rigorous design would instead build the prior from out-of-fold predictions obtained through cross-validation, as AMC-Axolotls did [7]. A visual trace of this effect appears in the training curves (Section 4, Figure 5, Figure 6 and Figure 7): the first stage M2.1, trained without any prior, converges almost identically to the single-stage M1, whereas M2.2, fed with the in-fold prior, saturates earlier, which is consistent with receiving an optimistic input on patients already seen during the prior’s training. The fact that even under this optimistic setting the cascade did not improve significantly over the single-stage network (Table 8; ΔDSC =0.001, p=0.583) makes the case against the added complexity stronger rather than weaker. On the balance of this evidence, doubling the training and inference cost by going from 5 to 7 channels does not appear justified on this dataset, although an out-of-fold cascade would be required to settle the question definitively.

### 5.3. CNN vs. Transformer Under Controlled Preprocessing

The M3 vs. M1 comparison keeps the input modalities, the preprocessing, the training and validation folds and the test set identical. It does not, however, isolate architecture in the strict sense: as detailed in Section 3 and Table 4, M1 and M3 also differ in optimiser (SGD versus AdamW), learning rate, patch size and physical field of view, spatial resampling, augmentation policy and model capacity (∼30 M versus 4.5 M parameters). These framework-dependent factors are coupled to each architecture and could each contribute to the observed gap. With that qualification, M1 shows higher point estimates than M3 on all four metrics, with the largest nominal separation in F1-lesion (7.60% vs. 2.30%) and DSC (0.210 vs. 0.163). This ordering is directionally consistent with the ISLES’24 leaderboard, where the three top submissions used CNN backbones and the best Transformer entry obtained DSC 0.068. However, on the present 15-patient cohort, none of these differences reached statistical significance (Table 8; DSC p=0.542, F1 p=0.109), so the result is best read as indicating that the local inductive bias of the convolutional model retained a practical advantage over the single Transformer evaluated *on this dataset and under this configuration*, rather than as evidence that CNNs are inherently superior to Transformers for volumetric stroke segmentation [6].

**Figure 11:**
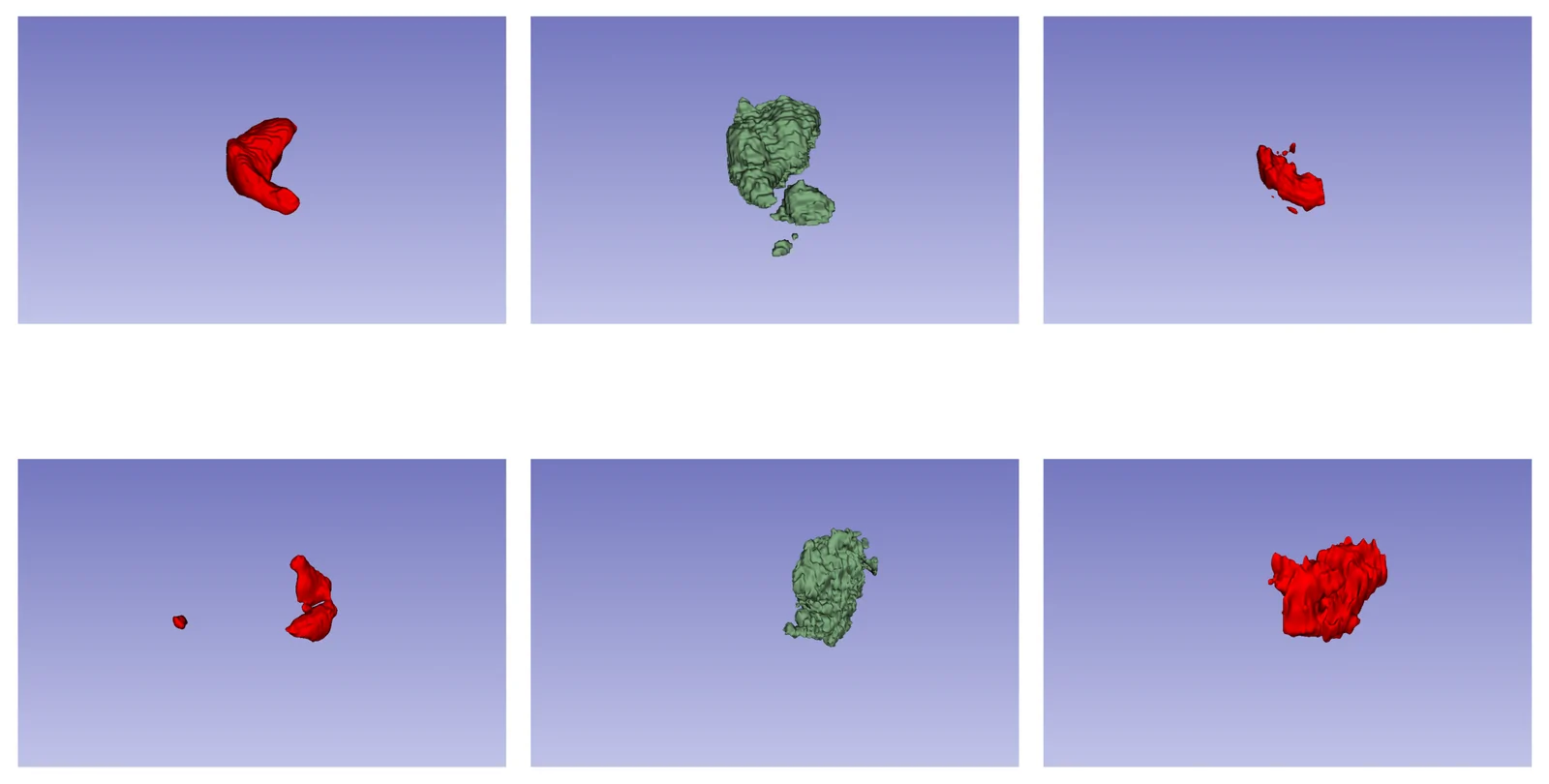
Three-dimensional rendering of M1 and M3 predictions on two representative cases from the held-out test set. Columns, from left to right: M1 prediction, ground-truth lesion mask, and M3 prediction. (**Top row**): sub-stroke0027 (M1 DSC 0.681, M3 DSC 0.338). (**Bottom row**): sub-stroke0082 (M1 DSC 0.271, M3 DSC 0.605). The ground-truth lesion mask is shown in green and the model prediction in red, overlaid on the skull-stripped CTA volume.

The patient-level distribution qualifies this conclusion. M1 and M3 trade victories case by case, M1 winning on sub-stroke0027 (DSC 0.681 vs. 0.338) and sub-stroke0148 (0.643 vs. 0.204), while M3 outperforms M1 on sub-stroke0082 (0.605 vs. 0.271) and sub-stroke0007 (0.423 vs. 0.064). Figure 11 renders one case from each subset in three dimensions. The asymmetry indicates that the architectures differ not only in average performance but also in the type of cases on which they succeed, suggesting that an ensemble of complementary architectures could improve worst-case behaviour.

A second comparison of great importance is worth highlighting: M3 vs. the Transformer entries of the official challenge. Despite training on a smaller pool with a single fold, M3 obtained DSC 0.163, against 0.139 of MIPLAB-PrediCTP (hybrid CNN-Transformer) and 0.068 of QuiiL/MoReT (pure Transformer) [7]. Regarding volumetric error, M3 (AVD 27.28 mL) is approximately three and six times lower than those of MIPLAB-PrediCTP (77.38 mL) and QuiiL/MoReT (168.06 mL). This is attributed to the fact that neither Transformer entry incorporated modality-specific clinical windowing, QuiiL/MoReT used min–max normalisation with Tmax thresholded at 9 s, and MIPLAB-PrediCTP applied baseline subtraction and Z-score normalisation [7,36].

These results do not necessarily show that Transformers are intrinsically inferior for volumetric stroke segmentation, as this type of architecture benefits from larger datasets, not just 150 cases [6]. What they do show is that, with small datasets, CNNs can outperform Transformers. This advantage could diminish with larger or more heterogeneous datasets, where the global receptive field of Transformer self-attention could be beneficial over CNNs.

### 5.4. Failure Modes: Small Lesions and Fragmentation

All four models share a low lesion-wise F1 (between 2.30% and 7.60%) and a high ALCD (between 5.40 and 7.27); even the winning Kurtlab submission obtained an F1 of 14.40% on the official test set. This is because the Dice loss function measures the global overlap between the prediction and the real mask at the voxel level, so a large 50 mL lesion contributes many more voxels than a small 1 mL one. As a result, the model learns that getting the large lesions wrong is penalised much more than the small ones, so it prioritises segmenting the large lesions well at the cost of worse results on the small ones [40]. Because of this, the F1-lesion metric was added to the ISLES’24 protocol, as it penalises each undetected lesion regardless of its size [7]. As future lines of work, loss functions that treat each lesion as an individual instance instead of counting voxels globally, or pipelines that first detect lesions and then segment them, could mitigate this problem.

### 5.5. Limitations and Clinical Translation

Several limitations bound the scope of these findings and should be read together with the paired statistical analysis of Section 4.3. First, the held-out test set comprises only 15 patients, and the paired Wilcoxon tests found no significant difference between any pair of models on any metric (Table 8); the study is therefore descriptive of this cohort and not powered to establish a systematic ranking, and the numerical orderings should be interpreted with corresponding caution. Second, each model was trained with a single data split, a single fold and a single random seed; repeating training across several seeds and splits, and reporting the mean and dispersion across runs, would separate genuine architectural effects from run-to-run variability and is a necessary step before any general claim can be made. Single-fold training also prevents best-fold selection or ensembling, both exploited by the top challenge submissions [8]. Third, the comparison between frameworks is not fully controlled: M1/M2 and M3 differ not only in architecture but also in optimiser, learning rate, patch size and physical field of view, spatial resampling, augmentation policy (full nnU-Net chain versus minimal MONAI chain) and model capacity, so the reported differences reflect the training strategy of each framework as a whole rather than architecture in isolation. Fourth, the in-fold mask generation discussed in Section 5.2 introduces an optimistic bias on the cascade behaviour that an out-of-fold, cross-validated prior would remove. Finally, ISLES’24 restricts inclusion to patients with successful endovascular reperfusion (TICI 2B or 3) and originates from two Central European academic centres [7], so generalisability to failed reperfusion, non-large-vessel-occlusion strokes or other geographies cannot be assumed, and no external multicentre validation was performed.

The performance regime reported here (DSC ≈ 0.22) falls below the threshold typically associated with clinical-grade segmentation [39], although this is due to the complexity of the predictive nature of the ISLES’24 task, the model must anticipate an infarct visible only on follow-up DWI between 2 and 9 days after admission, which places the upper bound of achievable performance well below conventional segmentation benchmarks [3,7]. Translation to a hospital workflow would require external multicentre validation, integration with PACS infrastructure, and inference times compatible with the therapeutic windows of acute stroke care [2]. Software qualifying as a medical device would also require regulatory clearance under the EU Medical Device Regulation or equivalent FDA pathways [44,45]. The present results should accordingly be read as a methodological contribution and not as a clinically deployable system.

## 6. Conclusions

In this work, three deep learning strategies are compared under controlled experimental conditions for automatic ischaemic stroke lesion segmentation on multimodal CTP imaging from the ISLES’24 dataset. The 133-patient training pool, the validation fold, the 15-patient held-out test set, and the preprocessing pipeline are identical across M1 (5-channel 3D nnU-Net), M2 (2→7-channel nnU-Net cascade) and M3 (SegFormer3D), while framework-dependent training settings (optimiser, learning rate, patch size, field of view, resampling, augmentation, and model capacity) remain coupled to each architecture and are treated as part of each compared strategy.

On the test set, the cascade M2.2 reaches the highest DSC (0.224); M1 obtains the lowest AVD (23.70 mL) and the highest lesion-wise F1 (7.60%); both cascade stages tie for the lowest ALCD (5.40); and M3 shows the lowest inter-patient standard deviation (0.178). Paired statistical testing over the 15 patients, however, found none of these differences to be significant, so the orderings describe this cohort rather than a general ranking. Within that limit, injecting the M2.1 prediction as the seventh channel is not justified by its cost–benefit ratio on this dataset, since the cascade does not improve significantly over the single-stage network even under an in-fold prior that favours it. In a purely contextual comparison, M3 reported better metrics than both Transformer entries of the official leaderboard (DSC 0.163 against 0.139 and 0.068) [7] despite being trained with less data, which is consistent with preprocessing quality being at least as influential as architectural choice, although the different training regimes prevent a definitive attribution. Finally, for this reduced-data setting, the local inductive bias of the convolutional models retained a practical advantage over the single Transformer architecture evaluated; establishing whether this holds in general would require matched training conditions, multiple seeds, and larger, multi-centre cohorts.

Four future lines of work can be highlighted according to the findings of this study. The first is that a possible increase in data, through the inclusion of patients from different clinical centres, so that the volume and heterogeneity of the training data can be scaled, could help evaluate whether Transformers under these data augmentation conditions achieve better results, closing the gap with CNNs. The next would be to determine whether replacing the DSC loss function with functions that penalise each lesion as an instance independently of its size [40] would help increase the low lesion-wise F1 observed in the models. Likewise, the option of heterogeneous ensembles that combine different inductive biases could be explored. Finally, the deployment of these models on embedded hardware through reduced-precision quantisation, measuring latency, memory, and metric degradation, opens a possible path to real-time inference in hospital environments.

## Figures and Tables

**Table 1:** Summary of the ISLES’24 public training set used in this work, including the distribution by clinical centre, the number of cases excluded during dataset preparation, and the final split into internal training/validation and held-out test subsets.

Property	Value
Total cases (official training set)	150
Centre #1 (Munich)	100
Centre #2 (Zurich)	50
Cases discarded during dataset preparation	2
Cases used in this work	148
Internal training/validation pool	133
Fold 0 training fold	106
Fold 0 validation fold	27
Held-out internal test set	15
Imaging modalities (input)	NCCT, CTA, CBF, CBV, MTT, Tmax
Reference standard	DWI-derived final infarct mask
Time gap (admission → DWI)	2–9 days
Inclusion criterion	TICI 2B or 3
Median voxel spacing	2.0×0.41×0.41 mm
Median volume size (after resampling)	∼70 × 410 × 336 voxels

**Table 2:** Modality-specific intensity windowing bounds applied during preprocessing. Voxels outside the reported ranges are clipped. HU denotes Hounsfield units for CT intensity values; mL/100 g/min denotes millilitres of blood per 100 g of tissue per minute for cerebral blood flow; mL/100 g denotes millilitres of blood per 100 g of tissue for cerebral blood volume; and s denotes seconds for temporal perfusion parameters.

Property	NCCT	CTA	CBF	CBV	MTT	Tmax
Window	[0,80]	[0,90]	[0,35]	[0,10]	[0,20]	[0,7]
Units	HU	HU	mL/100 g/min	mL/100 g	s	s

**Table 3:** nnU-Net 3d_fullres architecture configuration (identical backbone for M1, M2.1, M2.2; only input channels and fingerprint differ).

Property	Value
Network class	PlainConvUNet (3d_fullres)
Stages	6
Features per stage	[32,64,128,256,320,320]
Conv per stage (enc/dec)	2/2
Kernel sizes	[1 × 3 × 3,1 × 3 × 3,$\;3^{3},\;3^{3},\;3^{3},\;3^{3}$]
Strides	[1 × 1 × 1,1 × 2 × 2,1 × 2 × 2,$\;2^{3},\;2^{3},\;2^{3}$]
Normalisation	InstanceNorm3d (ε = $10^{-5}$, affine)
Non-linearity	Leaky ReLU
Patch size	40×224×192 voxels
Batch size	2
Target spacing	[2.0,0.41,0.41] mm (anisotropic)

**Table 4:** Training configuration. Values are shared across all nnU-Net stages (M1, M2.1, M2.2) unless stated otherwise.

Hyperparameter	nnU-Net (M1, M2.1, M2.2)	SegFormer3D (M3)
Architecture	PlainConvUNet 3d_fullres	SegFormer3D (default)
Input channels	5 (M1),2 (M2.1),7 (M2.2)	5
Parameters	∼30 M	4.5 M
Optimiser	SGD (Nesterov)	AdamW
LR ($\eta_{0}$)	$10^{-2}$	$10^{-4}$
Momentum	0.99	—
Weight decay	$3\times 10^{-5}$	$10^{-5}$
LR schedule	PolyLR (power 0.9)	PolyLR (power 0.9)
Epochs	250	250
Batch size	2	2 (train)/1 (val)
Patch size	40×224×192	96×96×96
Loss	Dice + CE	Dice + CE
Augmentation	nnU-Net default	MONAI minimal

**Table 5:** Performance of the four trained models on the 15-patient held-out test set. Values are mean (standard deviation) across patients. ↑: higher is better. ↓: lower is better.

Model	Arch.	DSC ↑	AVD (mL) ↓	F1-Lesion (%) ↑	ALCD ↓
M1	nnU-Net 3D, 5 ch.	0.210 (0.237)	23.70 (27.90)	7.60 (10.50)	6.20 (6.16)
M2.1	nnU-Net 3D, 2 ch.	0.223 (0.222)	27.69 (36.49)	4.60 (7.20)	5.40 (3.81)
M2.2	nnU-Net 3D, 7 ch.	0.224 (0.223)	28.38 (35.00)	5.30 (8.40)	5.40 (4.00)
M3	SegFormer3D, 5 ch.	0.163 (0.178)	27.28 (24.41)	2.30 (5.20)	7.27 (6.72)

**Table 6:** Pairwise comparisons between models. Δ values represent the change in each metric when moving from the first to the second model ($\Delta =M_{\mathrm{second}}-M_{\mathrm{first}}$). For DSC and F1, positive Δ favours the second model; for AVD and ALCD, negative Δ favours the second model.

Comparison	Isolated Factor	ΔDSC	ΔAVD (mL)	ΔF1 (pp)	ΔALCD
M3 → M1	Transformer → CNN (same 5-ch. input)	+0.047	−3.58	+5.30	−1.07
M2.1 → M2.2	Adding M2.1 mask as 7th input channel	+0.001	+0.69	+0.70	+0.00
M1 → M2.2	Single-stage → two-stage cascade	+0.014	+4.68	−2.30	−0.80

**Table 7:** Median and interquartile range (IQR, first–third quartile) of the four official metrics across the 15 held-out test patients, complementing the mean (standard deviation) of Table 5. ↑: higher is better. ↓: lower is better.

Model	DSC ↑	AVD (mL) ↓	F1-Lesion ↑	ALCD ↓
	Median [IQR]	Median [IQR]	Median [IQR]	Median [IQR]
M1	0.085 [0.037–0.335]	9.64 [5.36–36.80]	0.000 [0.000–0.182]	3.0 [1.5–9.0]
M2.2	0.200 [0.040–0.292]	16.44 [5.48–30.59]	0.000 [0.000–0.094]	4.0 [2.5–7.0]
M3	0.143 [0.012–0.206]	20.02 [10.16–37.84]	0.000 [0.000–0.000]	6.0 [3.5–9.0]

**Table 8:** Paired statistical comparison of the three model pairs over the 15 held-out test patients. Difference is computed as (first model − second model); 95% CI is a paired bootstrap interval for the mean difference. *p* is the two-sided exact Wilcoxon signed-rank test. For DSC, the Holm-adjusted *p* across the three pairs is also reported.

Comparison	Metric	Mean Diff. [95% CI]	Wilcoxon *p*	Holm *p* (DSC)
M1 vs. M3	DSC	+0.047 [−0.064, +0.163]	0.542	1.000
	AVD	−3.59 [−17.87, +12.09]	0.489	—
	F1	+0.053 [−0.005, +0.113]	0.109	—
	ALCD	−1.07 [−5.93, +3.27]	0.978	—
M1 vs. M2.2	DSC	−0.014 [−0.112, +0.100]	0.583	0.583
	AVD	−4.69 [−21.81, +11.37]	0.679	—
	F1	+0.023 [−0.043, +0.091]	0.652	—
	ALCD	+0.80 [−1.60, +3.20]	0.577	—
M2.2 vs. M3	DSC	+0.061 [−0.004, +0.137]	0.217	0.650
	AVD	+1.10 [−19.69, +21.40]	0.978	—
	F1	+0.030 [+0.001, +0.067]	0.094	—
	ALCD	−1.87 [−5.20, +1.00]	0.542	—

**Table 9:** Comparison between the four trained models and reference entries from the ISLES’24 leaderboard. Values are mean (standard deviation). ↑: higher is better; ↓: lower is better. ^†^ Reference entries: 150-patient training pool, multi-fold cross-validation, 100-patient hidden test set. ^‡^ This work: 133-patient training pool (15 additional patients reserved as held-out test set prior to any training), single fold, 15-patient internal test set. Architecture abbreviations—ResEnc L/M: nnU-Net v2 3D large/medium residual encoder; PlainConv: nnU-Net v2 3D PlainConvUNet; Cascade: two-stage cascaded nnU-Net; CNN-T 2D: 2D hybrid CNN-Transformer; MoReT: pure 3D Transformer.

Model/Team	Architecture	Train	Test	DSC ↑	AVD (mL) ↓	F1 (%) ↑	ALCD ↓
ISLES’24 official leaderboard ^†^							
Kurtlab (1st)	ResEnc L	150	100	0.285 (0.213)	21.23 (37.22)	14.40 (21.20)	7.18 (7.67)
AMC-Axolotls (2nd)	Cascade	150	100	0.263 (0.247)	21.31 (35.23)	14.94 (25.12)	7.66 (7.94)
Ninjas (3rd)	ResEnc M	150	100	0.255 (0.191)	26.29 (39.73)	9.92 (13.46)	5.98 (6.46)
MIPLAB-PrediCTP (9th)	CNN-T 2D	150	100	0.139 (0.158)	77.38 (68.23)	1.79 (3.47)	20.02 (12.72)
QuiiL/MoReT (9th)	MoReT 3D	150	100	0.068 (0.090)	168.06 (112.87)	1.12 (5.06)	7.11 (7.54)
This work ^‡^							
M1	PlainConv, 5ch	133	15	0.210 (0.237)	23.70 (27.90)	7.60 (10.50)	6.20 (6.16)
M2.1	PlainConv, 2ch	133	15	0.223 (0.222)	27.69 (36.49)	4.60 (7.20)	5.40 (3.81)
M2.2	PlainConv, 7ch	133	15	0.224 (0.223)	28.38 (35.00)	5.30 (8.40)	5.40 (4.00)
M3	SegFormer3D	133	15	0.163 (0.178)	27.28 (24.41)	2.30 (5.20)	7.27 (6.72)

**Table 10:** Per-patient metrics for the four cases shown in Figure 10. DSC: Dice similarity coefficient; AVD: absolute volume difference (mL); F1: lesion-wise F1-score; ALCD: absolute lesion count difference.

Patient	M1				M2.2				M3			
	DSC	AVD	F1	ALCD	DSC	AVD	F1	ALCD	DSC	AVD	F1	ALCD
sub-stroke0027	0.681	53.39	0.200	6	0.757	4.58	0.182	5	0.338	95.66	0.095	5
sub-stroke0082	0.271	97.27	0.000	2	0.589	82.27	0.200	2	0.605	20.02	0.182	3
sub-stroke0148	0.643	0.20	0.286	1	0.126	16.44	0.000	8	0.204	20.51	0.000	8
sub-stroke0007	0.064	57.69	0.063	16	0.287	2.88	0.000	16	0.423	36.76	0.061	15

## Data Availability

This study is based exclusively on the publicly available ISLES 2024 challenge dataset, which was released by the challenge organisers in anonymised form and can be obtained through the official ISLES 2024 challenge repository [7]. No new clinical data were collected for this work. The specific patient identifiers used for the training, validation, and held-out test partitions, together with the preprocessing and evaluation code required to reproduce the reported results, are described in Appendix A and are available from the corresponding author on reasonable request.

## Abbreviations

    The following abbreviations are used in this manuscript:

ADC	Apparent Diffusion Coefficient
ALCD	Absolute Lesion Count Difference
AVD	Absolute Volume Difference
BraTS	Brain Tumour Segmentation benchmark
CBF	Cerebral Blood Flow
CBV	Cerebral Blood Volume
CLAHE	Contrast-Limited Adaptive Histogram Equalisation
CNN	Convolutional Neural Network
CRF	Conditional Random Field
CT	Computed Tomography
CTA	CT Angiography
CTP	CT Perfusion
DSC	Dice Similarity Coefficient
DWI	Diffusion-Weighted Imaging
FCN	Fully Convolutional Network
IoU	Intersection over Union
ISLES	Ischaemic Stroke Lesion Segmentation
MICCAI	Medical Image Computing and Computer-Assisted Intervention
MLP	Multi-Layer Perceptron
MONAI	Medical Open Network for AI
MRI	Magnetic Resonance Imaging
mRS	modified Rankin Scale
MTT	Mean Transit Time
NCCT	Non-Contrast CT
NIHSS	National Institutes of Health Stroke Scale
nnU-Net	no-new-Net U-Net
PACS	Picture Archiving and Communication System
TICI	Thrombolysis in Cerebral Infarction
Tmax	Time-to-Maximum
ViT	Vision Transformer

## Appendix A. Reproducibility Details

To support reproducibility, this appendix collects the implementation details required to reproduce the reported results.

**Data and partitions.** All experiments use the public ISLES’24 training set [7]. Two cases were discarded during preparation (because of read errors in a reduced copy of the dataset), leaving 148 usable patients: a 133-patient training/validation pool and a 15-patient held-out test set. The held-out test patients are sub-stroke0007, 0008, 0009, 0026, 0027, 0033, 0037, 0049, 0082, 0084, 0086, 0089, 0097, 0148, and 0180. The test set was selected at random before any training and was never seen by any model. The 133-patient pool was split into a fold-0 training fold of 106 patients and a validation fold of 27 patients, identical for all models.

**Random seeds.** A fixed seed of 42 was used for the selection of the held-out split, for the calibration subsets where applicable, and for the bootstrap resampling described in Section 4.3. Each model was trained with a single fold and a single seed; multi-seed training is identified as future work in Section 5.5.

**Software and libraries.** Training and inference were run on Google Colab (NVIDIA Tesla T4). The environment used Python 3.12, PyTorch 2.8.0, nnU-Net v2 2.7.0 [5], MONAI 1.5.1 [38], scikit-image 0.26.0, SciPy 1.16.0, NumPy 2.0.2, nibabel 5.4.2, SimpleITK 2.5.2, and cc3d 3.24.0 [42]. SegFormer3D uses the public OSU-PCV Lab implementation [28] with the default configuration (in_channels = 5, num_classes = 2). Because the Colab environment was not version-pinned, these versions correspond to the Colab-preinstalled defaults (for Python, PyTorch, NumPy and SciPy) or to the latest stable PyPI releases available at the time of execution (March 2026); the SegFormer3D commit used is listed in the code accompanying the submission.

**Preprocessing.** The pipeline (Section 3.3) is applied identically to all models: morphological skull-stripping (threshold (0,120) HU, binary erosion for 3 iterations, largest 3D connected component with cc3d, dilation for 5 iterations, hole filling); modality-specific windowing (Table 2); per-channel min–max rescaling to [0,1]; 3D CLAHE (equalize_adapthist, clip limit 0.02, default kernel size); and foreground Z-score normalisation over brain-mask voxels, with the background set to zero. For M1/M2, the nnU-Net planner resamples to [2.0;0.41;0.41] mm; M3 operates on the native grid.

**Inference and post-processing.** nnU-Net models (M1, M2) use nnUNetv2_predict; M3 uses a sliding-window inferer ($96^{3}$ patches, overlap 0.5). Predictions are obtained as argmax over the class logits with no probability-threshold tuning and no morphological post-processing. For each model, the checkpoint with the highest validation Dice was selected (M1, epoch 90; M2.2, epoch 172; M3, epoch 167).

**Metrics.** The four official ISLES’24 metrics were computed with the panoptica implementation (lesion matching at IoU ≥20%, greedy one-to-one). Connected components use 26-neighbour 3D connectivity via cc3d [42]. Empty reference or prediction masks are handled following the ISLES’24 convention: a DSC of 1 when both masks are empty and 0 when only one is empty. Paired statistics (Section 4.3) use the two-sided exact Wilcoxon signed-rank test and a paired bootstrap (10,000 resamples, seed 42), with Holm correction for the primary DSC metric across the three model pairs.
